# Proteoform Detection Across 17,000 Single Nuclei From Differentiating Skin Keratinocytes

**DOI:** 10.1016/j.mcpro.2026.101621

**Published:** 2026-07-14

**Authors:** Vijaya Lakshmi Kanchustambham, Indira Pla, Tian Xu, Pei Su, Nickolas P. Fisher, Michael A.R. Hollas, Paulina Markiv, Uday Agarwal, Monika Lysakowska, Jared O. Kafader, Xiaomin Bao, Neil L. Kelleher

**Affiliations:** 1 Proteomics Center of Excellence, Chemistry of Life Processes Institute, Northwestern University, Evanston, Illinois, USA; 2 Departments of Molecular Biosciences, Chemistry, Chemical and Biological Engineering, The Feinberg School of Medicine, Northwestern University, Evanston, Illinois, USA; 3 Departments of Molecular Biosciences, Dermatology, Northwestern University, Illinois, USA; 4 Chan Zuckerberg Biohub Chicago, Chicago, Illinois, USA

**Keywords:** single-nucleus proteoform imaging mass spectrometry, single-cell proteomics, proteoform assignment score, human epidermal keratinocyte differentiation, individual ion mass spectrometry

## Abstract

Advances in single-nucleus RNA sequencing have demonstrated advantages over single-cell transcriptomics by enabling capture of dynamic cellular states and transcriptional activity in rare or difficult-to-isolate cell types. To achieve analogous enhancements in intact protein profiling, we applied single-cell proteoform imaging mass spectrometry (*scPiMS*) to nuclei for label-free, proteoform-resolved analysis of >10^4^ human skin cells. Using *scPiMS*, we surveyed ∼400 proteoforms across over 17,000 single nuclei isolated from human epidermal keratinocytes spanning undifferentiated (UD) proliferative to differentiated (DF) states, generating single-nucleus proteoform maps of epidermal differentiation. Proteoforms were assigned using intact mass tag matching to a reference proteoform library generated from bulk top-down LC-MS/MS analyses of UD and DF nuclei and subsequently applied to single-nucleus proteoform assignment scores. Unsupervised clustering of single-nucleus proteoform imaging mass spectrometry (*snPiMS*) data resolved 12 distinct proteoform-defined clusters spanning a continuum from UD proliferative states to terminal differentiation. A progenitor-enriched cluster (cluster 4) exhibited elevated high-mobility group-17, acetylated H2A/H2B variants, and H2A.Z, consistent with open and developmentally poised chromatin. An early differentiating population (cluster 3) was marked by histone H3 bearing H3K4 acetylation together with H3K9 monomethylation, indicative of transcriptional activation. Progressive histone H4 methylation (H4K20me1, H4K20me2, and H4K20me3) reflected cell cycle-coupled modification of newly synthesized H4 in progenitor-like nuclei prior to differentiation, corresponding to clusters 5, 2, and 8, respectively. Cluster 1 was enriched in H3 proteoforms bearing H3K4me3 together with H3K9me1, consistent with an active yet transcriptionally poised chromatin state. Differentiation-committed states (cluster 0) exhibited H3 proteoforms containing H3K4 and H3K9 acetylation together with H3K36me2 reflecting transcriptionally active chromatin. Bulk histone post-translational modification profiling corroborated these trends, with UD nuclei enriched in H3K9me2 and H4K20me1 and DF nuclei enriched in H4K20me2, H3K79me2, H3K27me2/3, and H3K36me3. Together, *snPiMS* uniquely resolves combinatorial histone proteoforms within individual nuclei, revealing a continuous chromatin trajectory across epidermal differentiation.

## Highlights

• First demonstration of proteoform profiling from single nuclei.
• Clustered cells based on single nuclei proteoform signatures.
• Detected changes in histone proteoforms during skin epidermal cell differentiation.
• Identified H4K20me1/2 proteoforms mark newly synthesized H4 during the cell-cycle.

## Graphical Abstract

**Figure:**
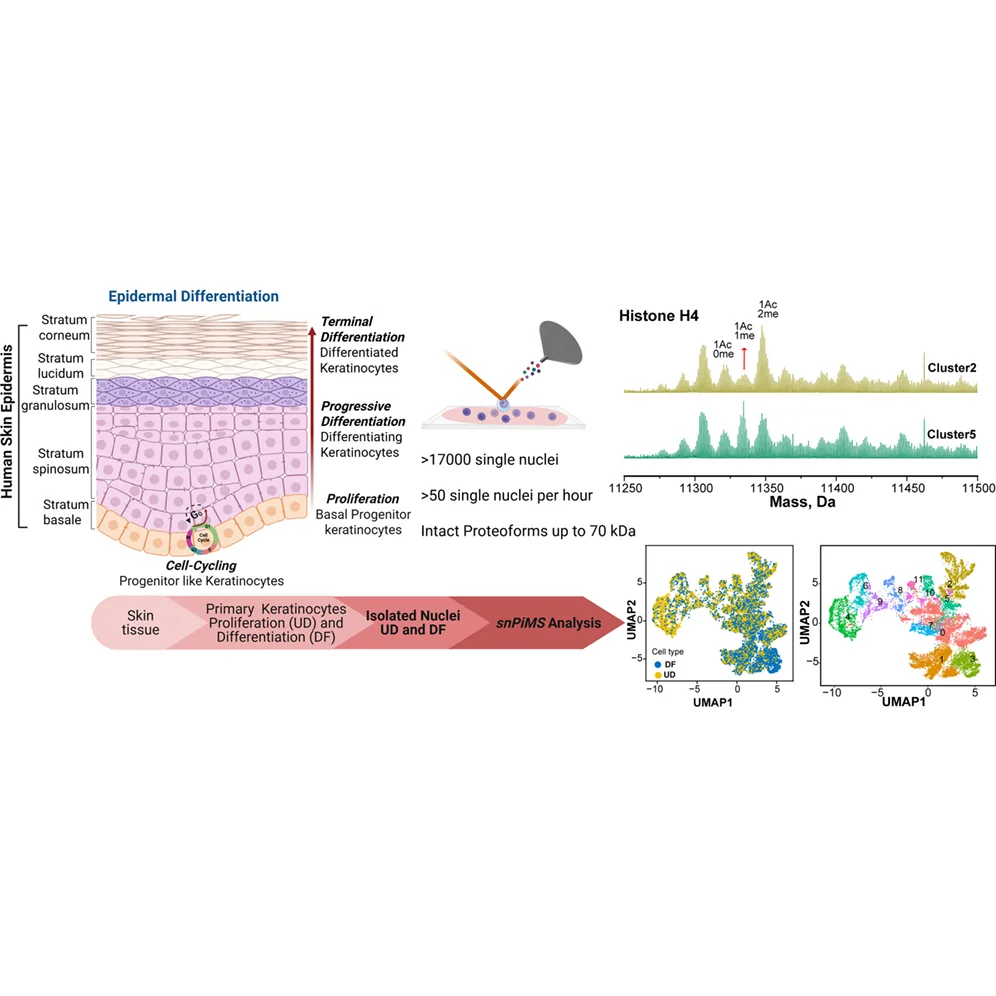

## In Brief

We introduce single-nucleus proteoform imaging mass spectrometry (*snPiMS*), a high-throughput platform for label-free profiling of intact proteoforms from individual nuclei. Applying *snPiMS* to >17,000 nuclei from differentiating human epidermal keratinocytes illuminates proteoform-level dynamics like the cell-cycle–linked methylation of histone H4 at K20. *snPiMS* establishes a scalable framework for single-cell proteomics and a new approach to single-cell biology in complex tissues.

Single-cell technologies have revolutionized biological research over the past decade, enabling high-resolution molecular profiling that uncovers the cellular heterogeneity present in biological systems. In contrast, traditional bulk sequencing methods average signals across heterogeneous cell populations, thereby masking critical cell-specific variations. Single-cell omics methodologies overcome this limitation by enabling the interrogation of individual cells, revealing diverse cell types, dynamic cellular states, and rare subpopulations. Next-generation sequencing platforms, particularly single-cell RNA sequencing (scRNA-seq), have further advanced this field by enabling quantitative measurement of thousands of transcripts and isoforms expressed in both model organisms and mammalian cells (1, 2, 3, 4, 5). Single-cell analysis led by high-throughput DNA sequencing and peptide mass spectrometry (MS) offers high-resolution insights into the genome, transcriptome, proteome, and epigenome of individual cells, providing new insights into cellular development, disease progression, and regulatory mechanisms (6, 7, 8, 9, 10, 11, 12). The integration of single-cell sequencing and MS-based omics offers unparalleled sensitivity and resolution, revealing how molecular heterogeneity shapes cell identity and function.

While scRNA-seq analysis informs transcriptomic diversity, proteoform-level information which ultimately governs cellular function remains largely unexplored. Proteoforms represent the distinct molecular forms of a protein originating from the same gene and arise from combinations of genetic variation, alternative splicing, and post-translational modifications (PTMs), including acetylation, phosphorylation, methylation, ubiquitination, and others (13, 14). As the primary functional effectors of the cell, proteoforms play a central role in defining cellular types and states (15, 16, 17, 18, 19, 20). Notably, mRNA abundance often shows poor correlation with corresponding protein levels at both population and single-cell scales (21, 22, 23, 24, 25, 26), highlighting the need for direct protein-level measurements (27). Although immunofluorescence-based techniques allow quantification of dozens of proteins per cell at single-cell resolution in tissues (28, 29, 30, 31, 32, 33, 34), advances in MS complement these capabilities. Single-cell proteomics (SCP) using MS now enables the quantification of protein abundance, protein-protein interactions, and cellular responses to physiological or pharmacological perturbations in both healthy and diseased states (35, 36, 37). However, SCP remains challenged by the wide dynamic range of proteins spanning from a few to millions of copies per cell (38, 39, 40, 41). Using “bottom-up” MS approaches, the field has made substantial progress in extending the scope of single-cell or single-shape analyses, including a recent effort to characterize protein transport at the level of single nucleus using peptide-centric workflows (42).

Combining the specificity of proteoform analysis with single-cell resolution remains challenging, particularly in converting tissues into suspensions of viable, intact cells using enzymatic dissociation or laser-capture microdissection. In this context, single-nucleus analyses have emerged as effective alternatives. Both single-nucleus transcriptomic (43, 44) and proteomic (42) profiling approaches offer distinct advantages when the isolation of intact single cells from complex tissues is difficult (43), and they are compatible with fresh, frozen, or even fixed samples. Single-nucleus RNA sequencing has been successfully applied to characterize diverse cell types in kidney (45), heart (46), and neuronal tissues (47, 48) among others (44, 49, 50, 51). At the protein level, single-nucleus measurements hold promise for capturing epigenetic and other nuclear processes that shape cell identity and state. However, the nucleus contains only ∼20 to 30% of the total cellular protein across various cell types and organisms (52, 53, 54). Total protein content per cell varies widely from as little as ∼42 pg in small follicular cells to ∼464 ± 35 pg in hepatocytes, with dendritic cell subtypes ranging between 64 ± 14 pg and 95 ± 25 pg per cell (52, 55). Using histones as a “proteomic ruler,” total nuclear protein content is generally estimated at <100 pg per nucleus, except in muscle cells, which can contain up to ∼675 pg of nuclear protein and exhibit 1000-fold higher total protein mass per cell (100 ng–1 μg) relative to typical somatic cells (52). A new frontier in single-nucleus biology lies in extending transcriptomic and peptide-based proteomic approaches to direct proteoform-level measurements. Unlike conventional “bottom-up” methods that infer proteins from peptides, intact-protein analysis enables direct detection of proteoforms, including the combinatorial PTMs involved in chromatin regulation, gene expression, and nuclear function.

Here, we demonstrate the application of individual ion mass spectrometry (I^2^MS) coupled with nano-desorption electrospray ionization (nano-DESI) for single-nucleus proteome profiling in primary human skin epidermal keratinocytes as they progress from progenitor to differentiated (DF) states. This approach enabled the detection of abundant intact proteoforms, partially revealing the molecular landscape of nuclear proteoform dynamics during epidermal differentiation. The use of I^2^MS, which is a variation of charge detection mass spectrometry (56), has been propulsive for detecting and characterizing intact proteoforms. Recent advances in this technology have reduced sample concentration by ∼1000-fold, facilitating detection of individual proteoforms from thin tissue sections (*i*.*e*., proteoform imaging mass spectrometry) (57, 58), single-cells (single-cell proteoform imaging mass spectrometry, *scPiMS*) (59, 60), and now, single-nuclei (single-nucleus proteoform mass spectrometry, *snPiMS*) (1, 61, 62, 63, 64). These techniques have been demonstrated to identify hundreds to thousands of proteoforms up to ∼80 kDa (57, 58).

In the present study, we extend single-nucleus analysis into the realm of proteoforms, showing high-throughput characterization of nuclei during human epidermal keratinocyte differentiation. The *snPiMS* workflow enabled highly sensitive detection of histone variants and other nuclear proteins, including their PTMs, associated with chromatin state changes. Integration of *snPiMS* data revealed distinct proteoform-defined clusters, capturing a continuum from proliferative progenitors through active cell-cycle phases to terminal differentiation. Collectively, this study establishes *snPiMS* as a powerful platform for cell-type and state characterization at the intact proteoform level, complementing single-nucleus transcriptomic frameworks and advancing the frontier of SCP.

## Experimental Procedures

### Keratinocyte Differentiation *In Vitro*

Primary human keratinocytes were isolated from surgically discarded human neonatal foreskin specimens of deidentified healthy donors, obtained from the Skin Biology & Diseases Resource-Based Center at Northwestern University, following the declaration of Helsinki. The use of primary keratinocytes in this research has been reviewed by the Institutional Review Board at Northwestern University, and it was determined to be not human subject research. Keratinocytes isolated from six deidentified donors were pooled for all the experiments conducted in this study, encompassing single nucleus PiMS, bulk top-down proteomics (LC-MS/MS), and bulk bottom-up proteomics (LC-MS/MS) to minimize donor-specific and batch-to-batch variability. Both undifferentiated (UD) and DF conditions were generated from the same pooled donor population. Keratinocytes were expanded by culturing in a 1:1 mixture of keratinocyte-SFM (Gibco #17-005-142) and Medium 154 (Gibco #M154500), maintained under subconfluent conditions to preserve the progenitor state. Keratinocyte differentiation was induced by seeding at 100% confluency with the addition of exogenous 1.2 mM CaCl_2_ for 4 days (Supplemental Fig. S1*A*) (61, 62).

### Nuclei Isolation

Nuclei isolation from both UD and DF keratinocytes was performed using hypotonic buffer to lyse the plasma membrane and release nuclei (62, 63). Keratinocytes were trypsinized, and the resulting cell pellets were resuspended in hypotonic nuclei extraction buffer (NEBA: 10 mM HEPES pH 7.4, 1.5 mM MgCl_2_, 10 mM KCl, with 1 × protease inhibitor EDTA-free from Roche). Cells were lysed by adding an equal volume of NEBB (NEBA supplemented with 0.4% NP-40: 10 mM HEPES pH 7.4, 1.5 mM MgCl_2_, 10 mM KCl, 0.4% NP-40, with 1 × protease inhibitor EDTA-free from Roche), followed by incubation on ice for 2 min. Nuclei were collected by brief centrifugation (5-15 s) and washed once with hypotonic buffer (1:1 mixture of NEBA and NEBB) to remove residual cytoplasmic components (Supplemental Fig. S1, *B* and *C*).

### Nuclei Preparation for *snPiMS* Analysis

Following extraction, nuclei were resuspended in a 1:1 mixture of NEBA and NEBB buffers. The nuclei concentration was determined using a hemocytometer under a light microscope (EVOS XL, ThermoFisher). Based on the count, the suspension was diluted to a working concentration of 1500–2500 nuclei per mL and transferred into a Nunc Lab-Tek II glass chamber slide (Thermo Fisher Scientific) featuring a CC2-treated growth surface with poly-lysine like adhesion properties (59). The nuclei suspension was drop-cast onto the chamber slide and centrifuged at 450*g* for 5 min to facilitate nucleus adherence to the glass surface. After centrifugation, the buffer was removed by gentle vacuum aspiration, and the nuclei were fixed with cold (−20 °C) 200-proof ethanol, then stored at −80 °C until analysis by *snPiMS*.

### High Contrast Microscopy

Prior to *snPiMS* analysis, whole slide brightfield images were acquired using a LionHeart Automated Microscope (BioTek, Agilent Technologies). Images were captured with a 4 × objective, maintaining a 13% overlap between adjacent fields of view, and subsequently stitched to generate a single composite image of the entire slide. The stitched images were processed and exported as.tiff files. Nuclei were manually counted in QuPath (version 0.5.1) using the point selection tool, and corresponding *x-y* coordinates were extracted for each nucleus and exported as a.csv file for downstream statistical modeling and *snPiMS* experiment programming using an in-house MATLAB script (16, 59).

### *snPiMS* Experiments

**Fig. 1:**
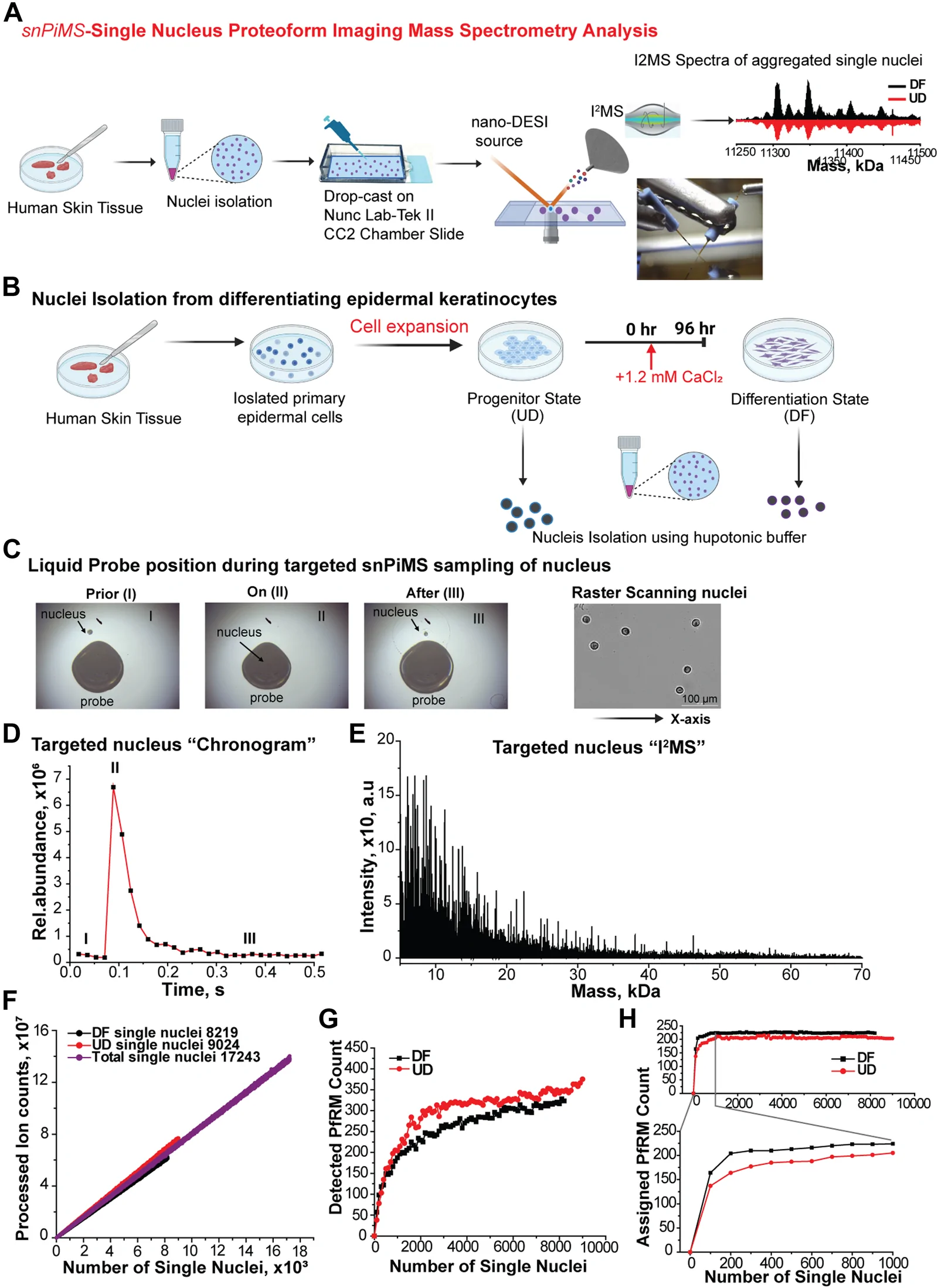
**Technical workflow diagram for profiling and assigning *snProteoforms*.***A*, panel (*A*) shows the single nuclei PiMS scanning approach. *B*, nuclei isolation and differentiation scheme of human neonatal foreskin epidermal keratinocytes. *C*, snapshots of the relative location of a cell (*C*) before (I), on (II), and after (III) analyte extraction by the moving liquid bridge (scale bar = 50 μm). *D* and *E*, the extraction profile of the targeted single nucleus (a “chronogram”) is shown at *left* (*D*), and (*E*) the targeted I^2^MS spectrum of single nucleus is shown on the *right*. *F*, collector’s curves for the number of charge-assigned ions in both DF (*black*) and UD (*red*) overlayed on to the total number of processed ions (*purple*), *G*, THRASH mass features detected viable isotopic distributions and corresponding IMT identified, *H*, proteoforms as a function of sampled single nuclei in both DF (*black*) and UD (*red*) *snPiMS* datasets. DF, differentiated; I^2^MS, individual ion mass spectrometry; IMT, intact mass tag; MS, mass spectrometry; nano-DESI, nano-desorption electrospray ionization; PiMS, proteoform imaging mass spectrometry; UD, undifferentiated.

*snPiMS* experiments were performed following a workflow adapted from previously established PiMS (57, 58) and *scPiMS* methodologies (59). The experimental setup consisted of a custom-built nano-DESI source configured for single-nucleus analysis, coupled to a Q Exactive Plus mass spectrometer (Thermo Fisher Scientific) customized for I^2^MS (64, 65). Detailed descriptions of the nano-DESI platform have been reported elsewhere (66, 67, 68). A schematic overview of the *snPiMS* workflow is shown in Figure 1. Briefly, the nano-DESI source comprises a flame-pulled fused silica primary capillary (OD 40 μm, ID 20 μm) positioned at a ∼90 to 120° angle relative to a secondary nanospray capillary (OD150 μm, ID 40 μm; Molex, MN), with the spray tip oriented toward the MS inlet. Probe alignment was monitored using a Dino-Lite digital microscope.

Slides containing nuclei were mounted on a motorized XYZ stage controlled by a custom LabVIEW program (available at https://github.com/LabLaskin) (66). During data acquisition, the slide was raster-scanned beneath the nano-DESI probe at a scan rate of 20 μm/s with a 200 μm line spacing. The working solvent was constantly delivered through the primary capillary at a flow rate of 0.5 μl/min using a syringe pump, forming a dynamic liquid bridge between the two capillaries and the sample surface. Analytes were extracted into this liquid junction and transferred through the nanospray capillary for electrospray ionization at the mass spectrometer inlet. Ion transfer into the mass spectrometer was guided by a Velos ion transfer tube with a 2 cm extension (Scientific Instrument Services, Inc.) to accommodate the geometry of the slide and nano-DESI probe. A denaturing solvent consisting of 5% acetic acid, 57.5% acetonitrile, and 37.5% water was used as the working solvent for all *snPiMS* experiments.

To minimize donor-specific variability, nuclei were isolated from pooled keratinocyte populations prior to differentiation into UD and DF states. While formal randomization was not performed, data acquisition was conducted in an alternating sequence (DF/UD/DF/UD) to minimize potential variations during the experiments.

### *snPiMS* Data Acquisition

To improve ion survival rates, the central electrode voltage of the Orbitrap analyzer was reduced to −1 kV. A higher-energy collisional dissociation gas pressure setting of 0.5 arbitrary units (high vacuum: 2 × 10^−5^ Torr; ultra-high vacuum pressure: <5 × 10^−11^ Torr) was used to minimize collision-induced ion decay while maintaining efficient trapping of intact protein ions within the Orbitrap analyzer (59, 65). Injection times were optimized between 200 and 700 ms to primarily capture individual ion events (Supplemental Fig. S2). The ion source conditions on the mass spectrometers were set as follows: electrospray ionization voltage: 3.1 kV; in-source collision-induced dissociation: 15 eV; S-Lens/Funnel RF level: 70%; capillary temperature: 340 °C. Additional data acquisition parameters that were adjusted as follows: mass range: 550 to 2500 *m/z*; AGC mode: fixed; enhanced Fourier transform: off; averaging: 0; microscans: 1. Time-domain data files were acquired at detected ion frequencies and saved as selective temporal overview of resonant ions (STORI) files for downstream processing.

### *snPiMS* Feature Selection and Data Analysis

We employed an in-house developed algorithm using MATLAB and a C^#^ script for feature extraction and selection of single nuclei-only proteoform profiling following methodologies previously described for *scPiMS* (16, 59). *snPiMS* raw data were acquired as continuous ion chronograms, representing signal traces from individual nuclei interspersed with background chemical noise originating from blank slide regions (Supplemental Fig. S3). Processed individual ion chronograms were subjected to automated peak picking with ion count threshold of 1500 and a minimum spatial separation of 350 μm between adjacent peaks (Supplemental Fig. S4). Multinuclei features were excluded based on the half-width-at-half-maximum (HWHM) of the chronogram peaks, where single-nucleus events were defined as peaks not exceeding 250 μm on the right side of the profile. To ensure accurate feature assignment, single-nucleus peak detections identified by the algorithm were further validated through manual nuclei counting and spatial coordinate mapping in QuPath (version 0.5.1) (59), enabling precise modeling of single-nucleus populations (Supplemental Fig. S5, *A–D*). The validated single-nucleus features were subsequently used for proteoform-level data analysis.

### I^2^MS Data Processing and Proteoform Mass Determination

During I^2^MS data processing, the quantized charge state (*z*) of each individual ion was determined from the slope of the induced image current, as calculated by STORI analysis. Charge assignments for each ion were statistically validated by comparing the slopes of isotopologue signals across different charge states throughout the dataset (65). The corresponding neutral masses of protein ions were calculated according to Equation 1:

(1) $$Mass=\left(\frac{m}{z}\times z\right)-\left(z\times M_{proton}\right)$$

The STORI files of the MS scans corresponding to single-nucleus features were extracted from the aggregated dataset and reprocessed to generate single-nucleus I^2^MS mass-domain spectra using STORIboard (Proteinaceous) (Supplemental Fig. S5*E*). Using this workflow, single nucleus from both progenitor (UD) and DF keratinocytes were analyzed to characterize proteoform-level differences across epidermal differentiation.

### Proteoform Identification Via Intact Mass Tags

Fragmentation-based characterization of proteoforms using charge-detection MS/MS has been previously demonstrated in tissue proteoform imaging mass spectrometry, also referred to as autoPiMS (57, 58). Our current *snPiMS* workflow is primarily focused on the sensitive detection of orbitrap-based in I^2^MS to determine the high-resolution of intact proteoform masses, as described above. Proteoform identities were subsequently assigned through intact mass tag (IMT) matching to a reference proteoform library generated from bulk top-down proteomic analysis using LC-MS/MS.

The summed mass-domain *snPiMS* spectra, containing a comprehensive library of detected ions, were converted to.mzML format and analyzed using a custom version of TDValidator (Proteinaceous) implemented with an MS^1^ IMT search function as described previously (58). Mass-domain spectra generated from single nucleus were internally calibrated using eight confidently identified proteoforms spanning the 10 to 60 kDa range (Supplemental Table S1). High-resolution analysis of the calibrated spectra using the Horn algorithm (THRASH) (69, 70) yielded approximately 400 distinct mass features (71, 72). The identities of these proteoforms were inferred from experimentally determined accurate intact masses, following previously established IMT-based proteoform identification strategies (69, 70, 73). To assign identities to these THRASH-extracted mass features, a reference database was generated *via* top-down proteomics of bulk nuclei isolated from UD and DF keratinocytes. Both capillary zone electrophoresis-MS/MS (CZE-MS/MS) and liquid chromatography-MS/MS (LC-MS/MS) approaches were employed to build this database using the same biological samples as the *snPiMS* experiments. Corresponding MS/MS fragmentation maps from these bulk top-down datasets were used to support proteoform assignments and validate cluster-associated markers identified in the *snPiMS* analysis. Complete experimental and data processing details for these top-down analyses are provided in the bulk top-down proteomics section (Supplemental Fig. S6 and Supplemental Table S2).

The IMT search was conducted with a mass tolerance of ±5 ppm for UD nuclei and ±3 ppm for DF nuclei (Supplemental Table S3). A wider tolerance (±5 ppm) was applied to the aggregated I^2^MS spectra of UD nuclei to improve the proteoform identification and to account for its lower signal-to-noise ratio and higher background, which affected the precision of peak centroid determination. The search space was considered for most common proteoform modifications, including methionine on/off, acetylation, phosphorylation, methylation, and hydroxylation, encompassing approximately 450 proteoforms for UD and 350 for DF datasets (Supplemental Table S7). Additional proteoform matches were validated through manual spectral inspection and annotation of putative PTMs observed within the IMT search space (Supplemental Table S8 and S9). Redundant entries were removed to yield a curated dataset for downstream single nucleus typing and clustering analyses (Supplemental Fig. S10). The final curated IMT proteoforms were also used for mass recalibration of individual ions in the I^2^MS datasets to correct for minor instrument drift across acquisition days. Calibrated technical replicates of *snPiMS* from DF and UD keratinocytes were subjected to proteoform assignments using the proteoform assignment score (*PAScore*) (59) and clustering *via* Seurat clustering analysis (https://satijalab.org/seurat/) (Supplemental Fig. S11).

### *PAScore* for *snProteoforms*

Using the THRASH algorithm (71) and IMT approach, isotopic distributions of proteoform features were extracted from the aggregated mass-domain spectrum obtained from approximately 17,000 single nuclei. To assign individual nucleus-level ion counts to candidate proteoform isotopic distributions in single nucleus proteoform (*snProteoform*) data, we applied an informatics framework called *PAScore*, previously developed in our laboratory for single cell proteoform assignments (16, 59). The *PAScore* is based on a mathematical model that integrates both the mass accuracy and relative abundance of the isotopologue masses of the ions to quantify the confidence of a match between experimentally observed ions and theoretical isotopic envelopes (Equation 2). This framework accommodates both abundant and sparsely distributed ion signals, yielding *PAScore* values ranging from 1 (100% confidence) to 0 (0% confidence). In matching a group of individual ions to a reference proteoform using the *PAScore* system, each ion was first matched to its isotopologue with a mass tolerance of ±0.3 Da. The probabilistic score was computed based on the inclusion–exclusion principle by Equation 2. These ion matches are scored and combined with all ion scores for a given proteoform to yield a single-nucleus proteoform score (*PAScore*) using the following equation:

(2) $$PAS=1-\prod_{k=1}^{n}\left[1-\left(P_{isotopologue\;}X\;P_{mass\;error}\right)\right]$$

where P_isotopologue_ represents the expected relative intensity of the matching isotopologue, and P_mass error_ denotes the probability score for the mass error estimated between the observed individual ion mass and the theoretical mass of the isotopologue. The P_mass error_ in *PAScore* utilizes the cumulative distribution function of a normal distribution with a mean and standard deviation determined by the theoretical mass and width of an isotopic peak, respectively (Equation 3).

(3) $$P\;mass\;error=\left\{ \begin{array}{c} 2\;X\;CDF\left(m_{ion}\right),m_{ion}<m_{iso}\\ 2\;X\;{\left[1-CDF\left(m_{ion}\right)\right]}_{r},m_{ion}>m_{iso} \end{array}\right.$$

The resulting *PAScore* values were aggregated across all matched ions to generate a single composite confidence score for each proteoform identified in the *snPiMS* dataset.

### *snPiMS* Cell Clustering

The ionCount matrix containing raw single-nucleus proteoform data was imported into RStudio (R version 4.3.2) for analysis using the Seurat package (version 5.2.1; http://www.satijalab.org/seurat) (74, 75, 76, 77). Quality control filtering was applied to remove low-quality nuclei. Only proteoforms detected in at least three nuclei were retained, and nuclei containing between 50 and 300 unique proteoforms were included for downstream analyses. Data normalization was performed using the LogNormalize method, in which feature (proteoform) counts for each nucleus were divided by the total ion counts for that nucleus, multiplied by a scaling factor (10,000 by default), and natural log–transformed using the *log1p*(*x*) function [log(1 + x)]. The top 200 most variable proteoforms were identified using the *vst* method implemented in the FindVariableFeatures function. The normalized expression matrix was subsequently scaled and centered using ScaleData function. Principal component analysis (PCA) was conducted on the 200 most variable proteoforms, followed by dimensionality reduction using both PCA and uniform manifold approximation and projection (UMAP) based on the first 15 principal components. A K-nearest neighbor graph was constructed using the top 15 PCA dimensions (Supplemental Fig. S12), and clustering was performed with a resolution of 0.2, determined empirically based on optimal cluster separation observed in UMAP visualizations. Because UD and DF nuclei were derived from the same pooled donor population, the observed clustering primarily reflects differentiation state rather than donor-specific variation, consistent with prior transcriptomic studies demonstrating high interdonor consistency during keratinocyte differentiation (78). Differentially expressed proteoforms among clusters and putative cell types were identified using a two-sided Wilcoxon rank-sum test implemented in the FindAllMarkers function. Thresholds were set to *logfc.threshold* = 0.25 and *min.pct* = 0.25 to ensure that proteoform features were present in at least 25% of nuclei within either cluster or cell type. Proteoforms with an adjusted *p*-value (false discovery rate) < 0.05 were considered significantly differentially expressed. Analysis scripts used for single nucleus proteoform clustering, statistical analysis, and visualization are publicly available at GitHub https://github.com/NCTDP/snPiMS_clustering.

## Results

### Single Nucleus Proteoform Analysis in Progenitor- or Differentiation-State Keratinocytes

We applied the *snPiMS* workflow (Fig. 1*A*) to characterize proteoform-level dynamics during the differentiation of primary human epidermal keratinocytes. To map the nuclear proteoform landscape across keratinocyte differentiation, *snPiMS* data were acquired from the single nuclei isolated from UD (progenitor state) and DF (calcium-induced, day 4) keratinocytes, respectively (Fig. 1*B*). A reflected light microscope equipped for high-magnification imaging (900×) was positioned beneath the glass slide to continuously monitor the nano-DESI probe trajectory and its interactions with drop-cast nuclei. Experimental parameters were optimized to enhance proteoform detection sensitivity, including washing conditions to reduce ion suppression and maximize proteoform ion collection (Supplemental Fig. S13 and S14). These optimizations collectively increased the number of detectable proteoforms while minimizing background noise.

To examine the kinetics of individual ion extraction, *snPiMS* was performed on a single, spatially targeted nucleus, allowing characterization of proteoform ion abundance as a function of time (Fig. 1, *C* and *D*). A low background signal was detected initially (time point I, 0-4 s), followed by a sharp increase in total ion current as the nano-DESI probe traversed the nucleus (time point II, 5-10 s). The signal returned to baseline after approximately five scans (∼5 s) and remained at this low level as the probe moved beyond the nuclear boundary (time point III, 11-30 s) as shown in Figure 1, *C* and *D* (Supplemental Fig S2, *A* and *B*). In contrast to previously reported ion extraction profiles for whole cells which exhibited an ∼8 s duration for proteoform ionization we observed a bit shorter ∼4 s extraction period for single nuclei. This reduction in sampling time is likely resulting from the smaller size and protein content of nuclei compared to intact cells. The extracted ion signals from each nucleus were converted directly into mass-domain spectra, yielding proteoform features with molecular masses up to ∼70 kDa (Fig. 1*E*).

To elevate rigor, a *snPiMS* study design was performed using a total of eight technical replicates for each of the two biological conditions. The optimized workflow achieved an average throughput of approximately 50 nuclei per hour, enabling the acquisition rate of over 1000 single-nucleus events per day. In total, proteoform profiles were generated from 17,243 single nuclei, 9024 nuclei isolated from the progenitor state (UD), and 8219 nuclei isolated from the DF state (Fig. 1*F*). On average, 6800 ions were detected per nucleus, with a variance of 2429 ions (1σ) (Supplemental Fig S5*D*). Collectively, over 138 million raw individual ions were measured over the course of the study, of which more than 50% were successfully charge-assigned, and subsequently analyzed for proteoform identification using the *PAScore* framework previously described for *scPiMS* (59).

From the aggregated dataset, over 400 proteoform isotopic distributions were detected. Using the THRASH algorithm, 236 isotopic mass distributions could be confidently matched to a reference proteoform in the IMT database. Collector’s curves for total detected mass distributions and corresponding IMT-matched proteoforms are shown in Figure 1, *G* and *H*, respectively. Both THRASH and IMT-based identifications plateaued rapidly at approximately 2000 and 200 nuclei, respectively. Although incremental gains in total mass feature detection were observed beyond 2000 nuclei, the depth of proteoform identification remained limited to approximately 200 proteoforms even when aggregating spectra from hundreds to thousands of nuclei, indicating that the depth and diversity of the proteoform detection represents the principal constraint in proteoform coverage.

### Proteoform Landscapes of Single Nuclei in Progenitor and DF Cell States

**Fig. 2:**
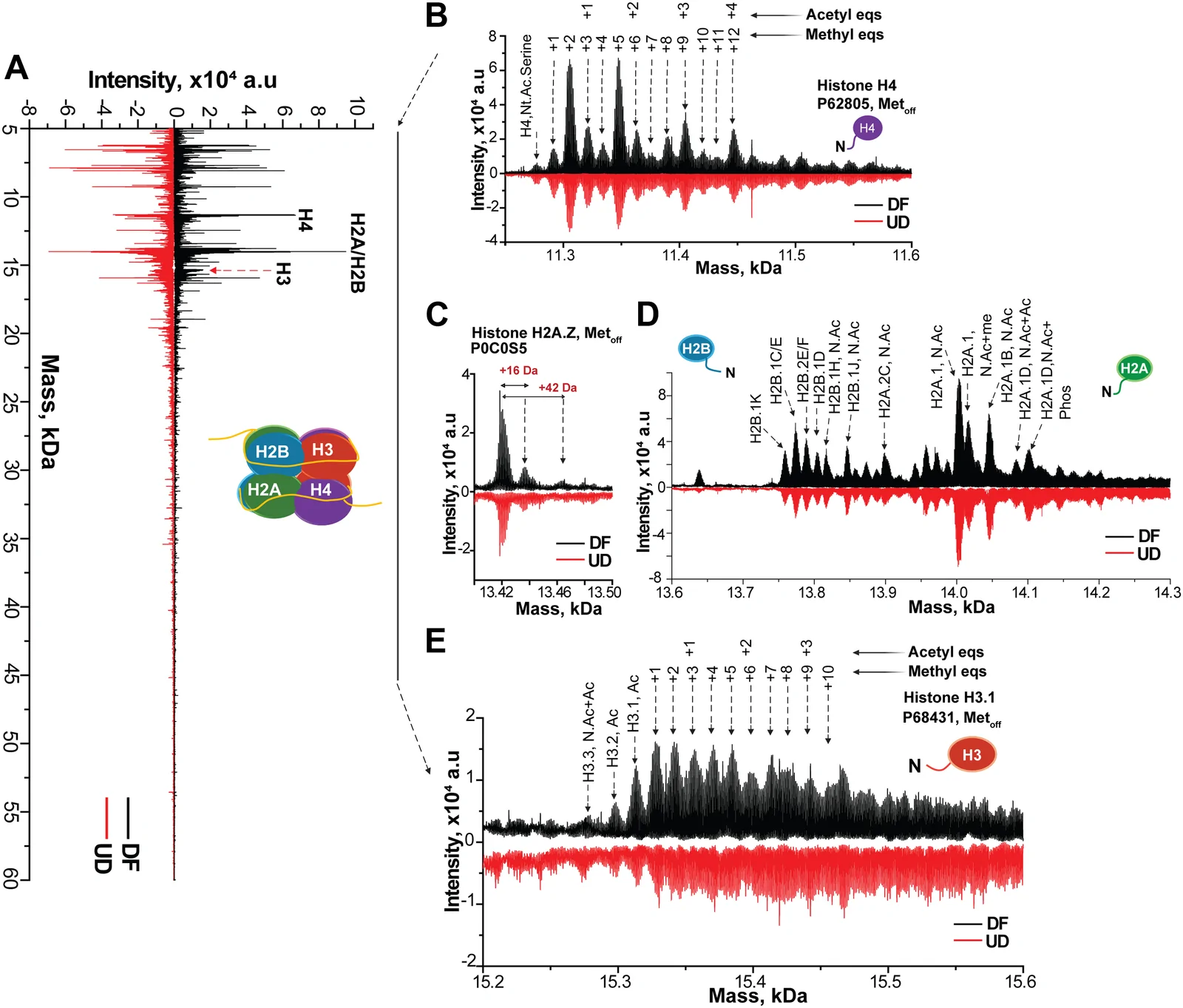
**Mass spectrum of aggregated keratinocyte nuclei.***A*, aggregated *snPiMS*-I^2^MS spectra of DF single nuclei (*black*) and UD single nuclei (*red*). *Inset* shows the MS range 11 to 16 kDa of the spectrum corresponding to histone H4, histone H2A & H2B, histone H3. *B*, expanded view of I^2^MS spectrum of histone H4 proteoforms including methylation and acetylation equivalents. *C*, expanded view of H2A.Z with a Δmass = +42 Da shift corresponding to acetylation modification. *D*, expanded view of H2B and H2A proteoforms. *E*, expanded view of H3 proteoforms with methylation and acetylation equivalents. DF, differentiated; I^2^MS, individual ion mass spectrometry; MS, mass spectrometry; snPiMS,single-nucleus proteoform imaging mass spectrometry; UD, undifferentiated.

To compare nuclear proteoform profiles between UD and DF keratinocytes, ion signals from progenitor state (9024 single nuclei, UD) and DF state (8219 single nuclei) were aggregated to generate global mass spectra for each condition (Fig. 2*A*). The resulting I^2^MS spectra revealed distinct yet broadly conserved proteoform profiles across both sample types. In the mass range 11 to 16 kDa (Fig. 2, *B*–*E*), corresponding to the core histones and their variants: H4, H3 (H3.1, H3.2, H3.3), H2B, H2A, H2A.Z, and H2AX, respectively. The aggregated I^2^MS spectra displayed comparable overall patterns of methylation and acetylation marks of core histones H4, H2A, H2B, and H3 between UD and DF nuclei, with only subtle shifts in PTM distributions observed across differentiation. We identified linker histone H1 within the 20 to 42 kDa mass range, as well as a set of nonhistone chromatin proteins from the high-mobility group (HMG) superfamily including HMGN1, HMGN2, HMGN3, HMGN4, and HMGA1 detected in the 8 to 11 kDa mass range (Supplemental Figs S9 and S21) (79, 80). In addition to histones, abundant proteoforms including large ribosomal subunits (RL23, RL24, RL26, RL28, RL30, RL34, RL35A, RL37, RL37A, RL38, RL39, and RL40) and small ribosomal subunits (RS10, RS11, RS14, RS15, RS16, RS18, RS19, RS20, RS21, RS23, RS26, RS27, RS28, and RS30) were detected in the single-nucleus proteome (Supplemental Figs S9). The presence of ribosomal subunit proteoforms in the *snPiMS* dataset likely reflects that either they are synthesized in the cytoplasm, imported back to the nucleus to assemble with rRNA in the nucleolus (81), or trace cytoplasmic carryover during nuclei isolation. Consequently, ribosomal subunit proteoforms were removed from the reference database prior to downstream proteoform clustering and differential expression analyses to maintain nuclear proteome specificity. To assess their impact, we additionally performed clustering analyses including ribosomal proteoforms (Supplemental Figs S15–S17); however, the main analyses presented here focus on clustering results obtained after their exclusion. In contrast, proteoforms above ∼45 kDa were less frequently identified, primarily due to limited database coverage for intact mass-based matching.

### Assignment Scores for Proteoforms in Single Nuclei

**Fig. 3:**
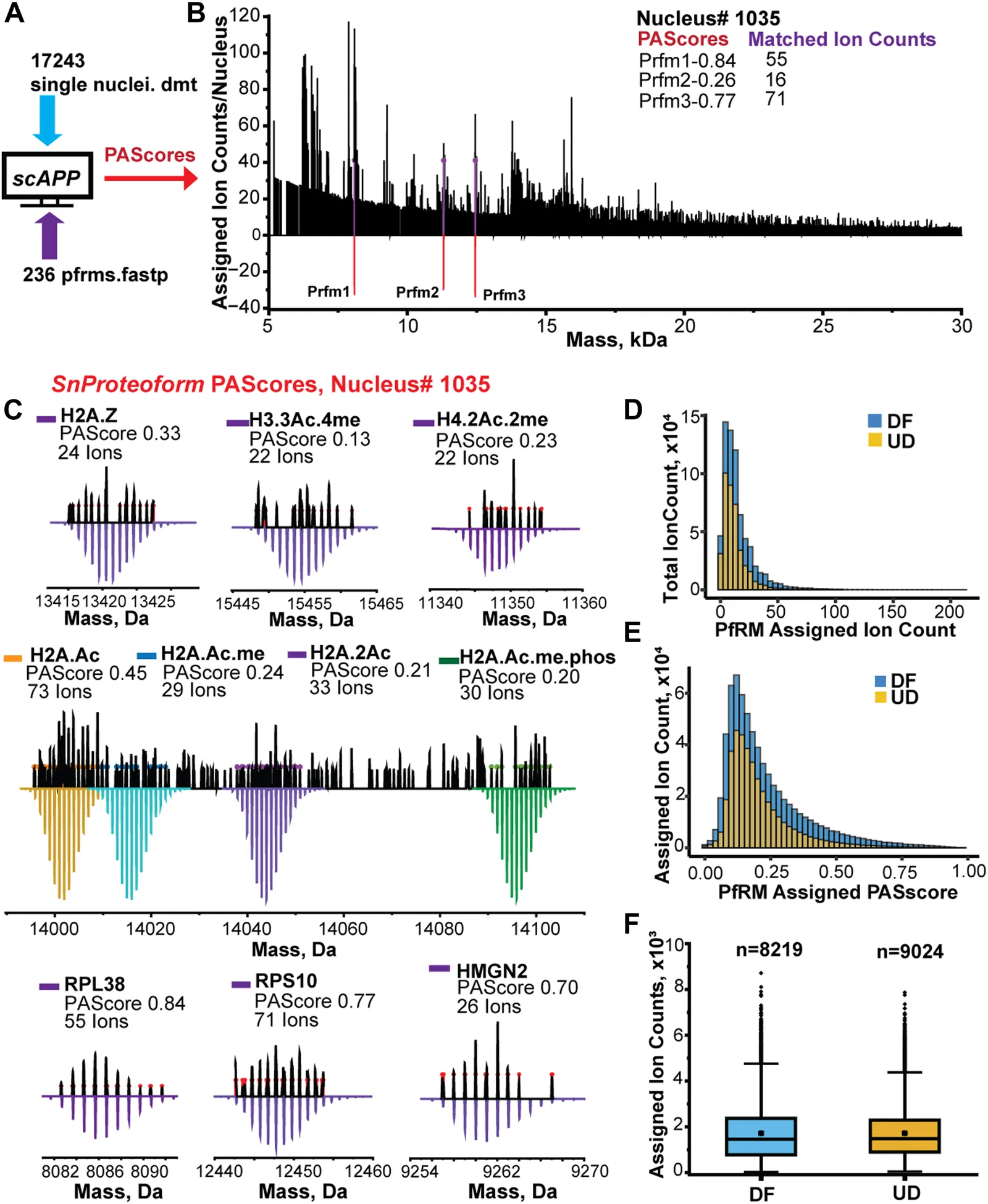
***snProteoforms* and proteoform assignment scores (*PAScores*).***A* and *B*, schematic workflow of single-cell proteoform analysis using *scAPP,* illustrating *PAScores* for sampled nucleus #1035. C, ten examples of single nuclei *PAScores* illustrating individual proteoforms, theoretical isotopic distribution (*bottom*), single nucleus spectrum (*black trace* on the *top*), and *snProteoform* (dots with droplines in the *middle*, *red*). *D*, distribution of the total number of ions assigned to proteoforms and *E*, distribution of *PAScores* across UD and DF single nuclei data. *F*, box- and whisker- plots for the assigned proteoform ion counts per nucleus in DF and UD single nuclei data. DF, differentiated; UD, undifferentiated.

To visualize proteoform patterns distinguishing UD from DF nucleus, each detected ion was parsed back to the single nucleus from which it was observed (Fig. 3). For every nucleus, each ion was characterized by its *m/z*, assigned charge state, and computed neutral mass, allowing for targeted interrogation of proteoform ion signals specific to individual or subsets of nuclei. An in-house single-cell application (*scAPP*) was used to parse and aggregate single ions into bins indexed to their respective nucleus, generating single-nucleus specific mass spectra; for example, the spectrum from nucleus #1035 contains ∼4000 individual ions (Fig. 3*A*). The *scAPP* pipeline then applied the *PAScore* (59) to assign single ions to matching *snProteoforms* (Fig. 3, *B* and *C*; see Experimental Procedures for details). Using *PAScore*-based assignments, we estimated an average of approximately 200 *snProteoforms* per nucleus with nonzero *PAScores*, while ∼130 *snProteoforms* per nucleus were retained using a *PAScore* cutoff of 0.005, corresponding to an estimated false discovery rate of 5% (59). Representative proteoforms, their *PAScores*, and the number of assigned ions per isotopic distribution are shown for the histone and nucleosome binding proteins from nucleus #1035 (Fig. 3*C*). On average, ∼10 to 15 single ions were assigned to each *snProteoform*. Typical *PAScores* ranged from 0.2 to 0.95, with higher scores reflecting greater confidence in assignment, particularly for abundant proteoforms. Although total ion counts were slightly higher for DF nuclei relative to UD nuclei (Fig. 3, *D* and *E*), the number of ions assigned to specific proteoforms was comparable between the two conditions (Fig. 3*F*). Although more nuclei were sampled in the UD condition, fewer ions were assigned to mass features relative to DF, consistent with prior observations of lower signal-to-noise in UD nuclei impacting PAScore-based assignments.

### Proteoform-Based Single-Nucleus Clustering and Cell-State Composition

**Fig. 4:**
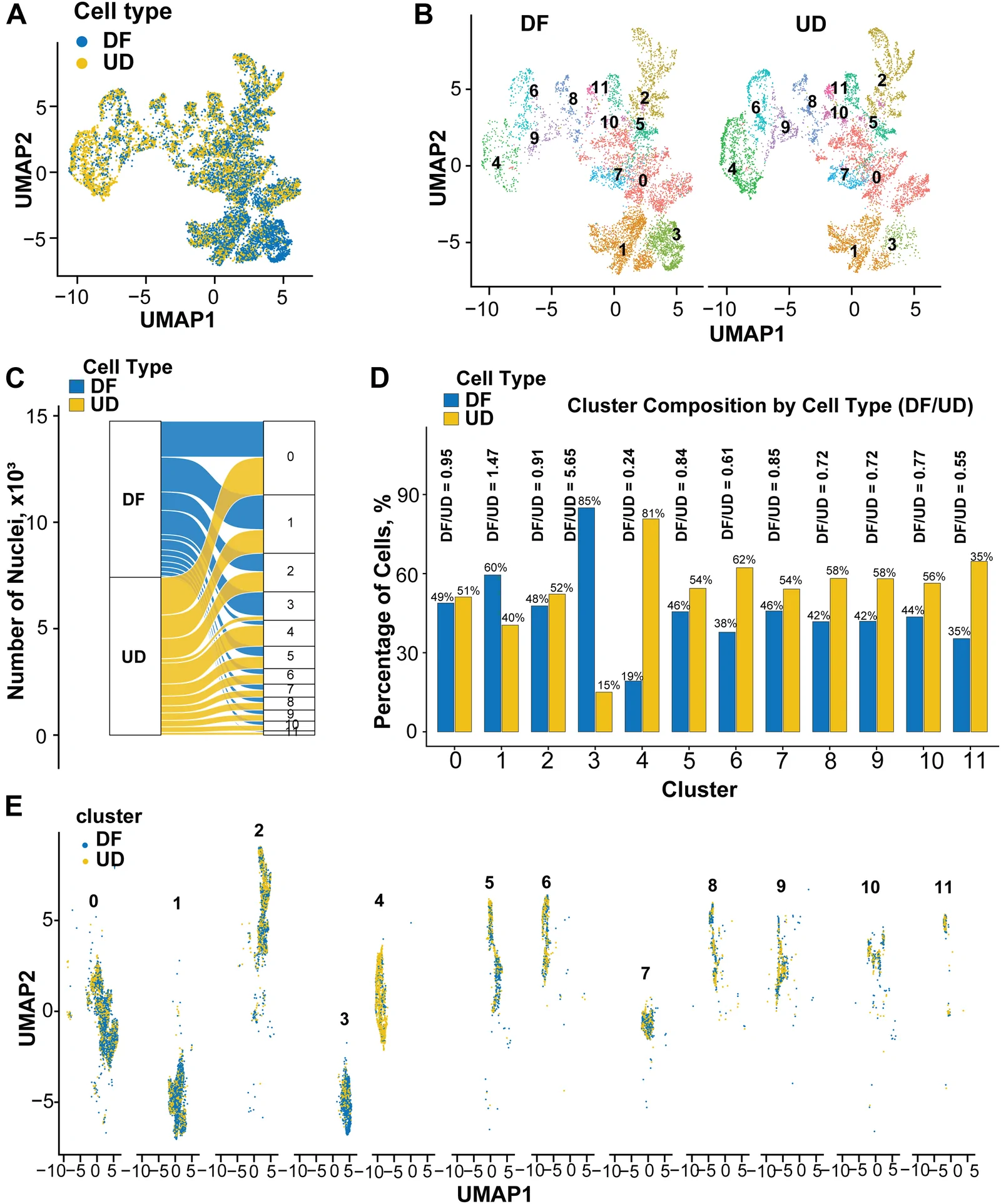
**Single-nucleus clustering and integrated analysis of undifferentiated and differentiated keratinocytes.***A*, UMAP visualization of 17,243 single nuclei profiled by single-nucleus proteoform imaging mass spectrometry (snPiMS), following normalization and integration using Seurat. Each dot represents an individual nucleus, color-coded by cell type (*blue*, differentiated [DF]; *yellow*, undifferentiated [UD]). The integrated dataset reveals well-mixed yet distinct nuclear populations, reflecting successful batch correction and preservation of biological heterogeneity between UD and DF keratinocytes. *B*, UMAP visualization of the same 17,243 nuclei showing proteoform-defined clustering into 12 distinct single-nucleus clusters (0–11) based on integrated snPiMS data. Each dot represents an individual nucleus colored by cluster identity. The UMAPs are also displayed separately by cell type (*left*, DF; *right*, UD). *C*, Sankey plot illustrating the relative distribution and the flow of nuclei between UD and DF enriched clusters, capturing the progressive chromatin transitions and cell-state continuum underlying the keratinocyte differentiation trajectory. *D*, cluster composition by cell type, showing the relative proportions of DF and UD nuclei within each cluster. *E*, UMAP visualization of all 12 proteoform-defined clusters (0–11) colored by cell type (*blue*, DF; *yellow*, UD), showing the relative enrichment of UD and DF nuclei within individual clusters, illustrating how cell-type composition aligns with the proteoform-defined chromatin states along the keratinocyte differentiation continuum. UMAP, uniform manifold approximation and projection.

Integration and normalization of all *snPiMS* datasets from UD and DF keratinocyte nuclei identified 192 highly variable nuclear-specific proteoform features for downstream analysis. Cytoplasmic and non-nuclear proteoforms, including ribosomal and structural proteins, were excluded to ensure nuclear specificity. Data from 17 technical replicates were integrated using *Seurat* with stringent quality-control thresholds to minimize batch effects and biological variability associated with cell-cycle and differentiation heterogeneity. Dimensionality reduction was performed using PCA followed by UMAP, resulting in 17,243 nuclei forming well-integrated yet distinct populations corresponding to UD and DF keratinocytes (Fig. 4*A*, blue, DF; yellow, UD). The integrated dataset demonstrated effective batch correction while preserving biologically relevant heterogeneity between cell states (Supplemental Fig. S12). Although UD and DF nuclei were obtained from the same batch of primary keratinocytes pooled from multiple donors, the observed clustering is driven primarily by differentiation state rather than interdonor variability, consistent with transcriptomic evidence of high concordance across donors (78). Proteoform-based clustering of the same nuclei revealed 12 distinct nuclear clusters (0-11) defined by their characteristic proteoform signatures (Fig. 4*A*). Separate visualization of DF and UD nuclei highlighted distinct yet continuous transitions across proteoform-defined cell states (Fig. 4*B*), representing progressive chromatin remodeling and cellular differentiation along the keratinocyte trajectory. A Sankey plot illustrated the relative distribution and flow of nuclei between UD and DF-enriched clusters, capturing the progressive chromatin transitions and cell-state continuum underlying the keratinocyte differentiation trajectory (Fig. 4*C*).

Consistently, a combined UMAP visualization of all 12 clusters colored by cell type (blue, DF; yellow, UD) revealed the differential enrichment of UD and DF nuclei within individual clusters, aligning proteoform-defined chromatin states with the cellular differentiation continuum (Fig. 4*D*). Each cluster contained nuclei from both UD and DF conditions, but their relative proportions varied substantially (Fig. 4*E*). Cluster 0 was essentially balanced (DF/UD ≈ 0.95), while cluster 1 and cluster 3 were DF-skewed, with cluster 3 showing the strongest DF predominance (DF/UD ≈ 5.64) and cluster 1 moderately DF-enriched (≈1.47). In contrast, Cluster 4 was markedly UD-dominant (DF/UD ≈ 0.24), containing the largest population of UD basal nuclei. Clusters 2 and 5 to 11 exhibited variable degrees of UD enrichment (DF/UD ≈ 0.55-0.85), corresponding to intermediate and postmitotic differentiating nuclei (Supplemental Table S4). Collectively, these distributions delineate a continuous proteoform-defined progression from basal proliferative progenitor (cluster 4), through early differentiation (clusters 0-3), and other continuum of cells during differentiation (clusters 5-11). Clusters 4 and 3 represent the cleanest molecular signatures of UD *versus* DF basal keratinocytes, respectively.

### Proteoform Signatures Define Distinct Keratinocyte Cell States Across Differentiation

To further delineate the molecular distinctions underlying these clusters, differential proteoform expression analysis was performed across all nuclei to identify proteoform signatures that define UD *versus* DF cell states and the potential proteoform patterns associated with epidermal keratinocyte differentiation. Using the Wilcoxon rank-sum test (adjusted *p* < 0.05, |log_2_FC| > 1), we identified 170 unique proteoforms significantly upregulated in at least one cluster (Supplemental Table S5). The resulting proteoform profiles capture coordinated histone PTMs, including acetylation, monomethylation, dimethylation, and trimethylation, (me1/me2/me3) as well as phosphorylation (p), which together correspond to distinct cellular states (82). Among known histone PTMs, lysine methylation on histones H3 and H4 plays well-established roles in regulating chromatin structure and function, with both site specificity and methylation state (monomethylation, dimethylation, or trimethylation) dictating distinct regulatory outcomes (83, 84, 85, 86).

**Fig. 5:**
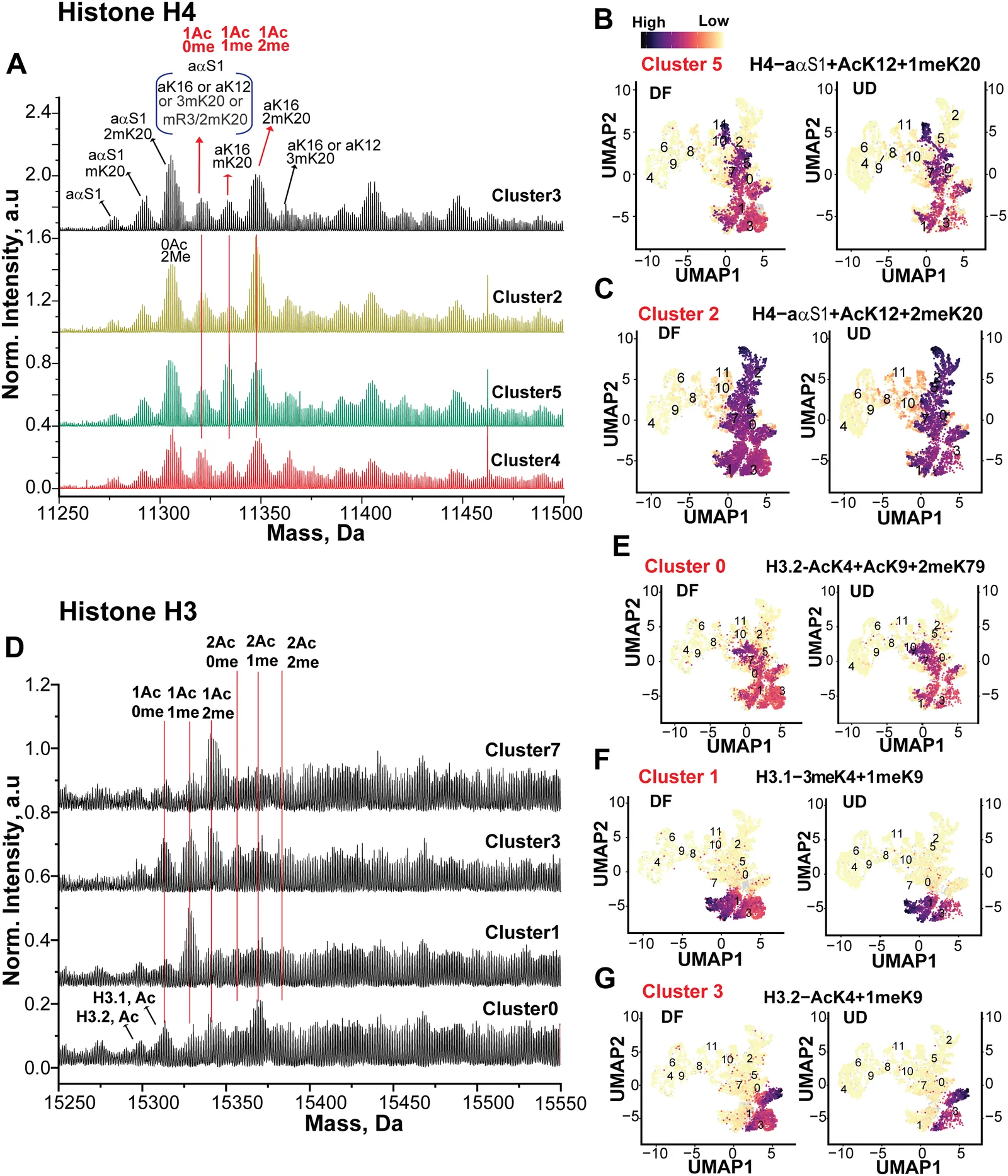
**Aggregated I^2^MS spectra reconstructed for each cluster derived from single-nucleus PiMS (*snPiMS*) profiles.***A*, cluster-specific H4 proteoform expression patterns revealed by *snPiMS*. Progressive methylation of N-terminally acetylated H4 in the mass range 11,250 to 11500 Da, including lysine K12 or K16 acetylation, across differentiation states histone H4 is α-N-terminally acetylated at serine 1; PTMs are denoted as a-acetyl, m (monomethyl), 2m (dimethyl), and 3m (trimethyl). Methylation and acetylation are also denoted as Me and Ac. *B* and *C*, UMAP visualization of single nuclei highlighting the top proteoform features most highly expressed in clusters 5 and 2, derived from integrated *snPiMS* data. *D*, cluster-specific H3 proteoform expression patterns revealed by *snPiMS.* Combinatorial acetylation and methylation patterns of histone H3 enriched in differentiation-committed clusters in the mass range 15,250 to 15550 Da. *E*–*G*, UMAP visualization of single nuclei highlighting the top proteoform features most highly expressed in clusters 0 to 3, derived from integrated *snPiMS* data. Each panel displays the relative expression intensity of representative proteoforms within the corresponding cluster, revealing distinct chromatin modification landscapes and proteoform signatures that define undifferentiated (UD), differentiating, and differentiated (DF) keratinocyte states along the epidermal lineage trajectory. I^2^MS, individual ion mass spectrometry; PTM, post-translational modification; UMAP, uniform manifold approximation and projection.

Collectively, these covalent histone modifications drive dynamic changes in chromatin architecture that are essential for the regulation of fundamental biological processes, including cell states and development. Single-nucleus clustering revealed distinct proteoform signatures that map onto the epidermal keratinocyte differentiation hierarchy. Figure 5*A* shows aggregated I^2^MS spectra of histone H4 in the 11,250-11,500 Da mass range, reconstructed for clusters corresponding to progenitor cells–enriched cluster 4 (red), differentiating cell–enriched cluster 3 (black), and cell-cycle coupled progenitor state–enriched clusters 5 (cyan), and 2 (dark yellow) that span the transition from proliferative progenitor state toward early differentiation. The spacing between adjacent spectral features is approximately 14 Da, reflecting the presence of abundant PTMs, including monomethylation (+14 Da), dimethylation (+28 Da), trimethylation (+42 Da), and acetylation (+42 Da). Although acetylation neutralizes the positive charge of lysine residues whereas trimethylation retains it, the global charge state of intact histones does not change sufficiently to distinguish these modifications in I^2^MS/charge detection mass spectrometry measurements. In addition, acetylation (+42.0106 Da) and trimethylation (+42.0469 Da) differ by only 0.0364 Da and are effectively isobaric at the intact proteoform level within the resolving capability of the current *snPiMS* dataset. Consequently, these PTMs cannot be unambiguously distinguished based solely on intact-mass measurements without fragmentation.

All detected H4 proteoforms were Nα-acetylated at Ser1, consistent with canonical histone H4 processing (H4Nαac). Accordingly, the baseline (unmodified) form referenced in this study corresponds to the Nα-acetylated H4 backbone (H4Nαac), and the PTMs are indicated using one-letter code “me1”, “me2”, and “me3”, for monomethylation, dimethylation, and trimethylation, respectively, and acetylation denoted as “ac” (83). Methylation, acetylation, and phosphorylation are also abbreviated as me and ac in spectral annotations. Spectra for each cluster are shown following intensity normalization to the maximum peak intensity within that cluster.

Cluster 5 exhibits an approximately 2-fold increase in monomethylated histone H4 proteoforms corresponding to Nα-acetylated H4 with K20me1 and acetylation localized to either K12 or K16 (H4NαacK20me1K12ac/K16ac), relative to the unmethylated species (Supplemental Fig. S18*A*). In contrast, cluster 2 exhibits an approximately 3-fold increase in dimethylated H4 proteoforms corresponding to Nα-acetylated H4 with K20me2 and acetylation at either K12 or K16 (H4NαacK20me2K12ac/K16ac), relative to the corresponding K20-unmethylated form (Supplemental Fig. S18*B*).

Due to reliance on intact-mass measurements with IMT matching to a top-down LC-MS/MS proteoform database, some modification sites cannot be uniquely localized, particularly acetylation at K12 *versus* K16 and methylation on histone H4, which may represent isomeric species. Accordingly, these assignments correspond to Level 2A–3A proteoforms (87). In addition, fragmentation patterns from top-down LC-MS/MS data (Supplemental Fig. S18) are most consistent with methylation at H4K20, with additional evidence for H4R3 methylation, while acetylation is observed at canonical sites (K5, K8, K12, and K16). Although bottom-up MRM data further support H4K20 methylation, alternative site assignments (*e.g*., K16 or R17) cannot be fully excluded due to limitations in precise site localization. Together, these results support H4K20 as the predominant methylation site while acknowledging residual ambiguity arising from isomeric proteoforms.

Across clusters, the observed changes in global abundance for H4K20 methylation are consistent with progressive methylation of newly synthesized H4 reported previously (82, 83). Accordingly, the transition from H4K20me1 to H4K20me2 observed between clusters 5 and 2 reflects cell-cycle coupled chromatin maturation occurring in progenitor-like nuclei prior to cell-cycle exit and differentiation. Consistent with the aggregated I^2^MS spectra, UMAP feature-expression plots further support enrichment of these H4K20-methylated proteoforms in clusters 5 and 2 (Fig. 5, *B* and *C*), supporting their contribution to proteoform defined clustering in the *snPiMS* analysis.

Figure 5*D* shows aggregated I^2^MS spectra of histone H3 in the 15,250–15,550 Da mass range, reconstructed for clusters corresponding to differentiating cells (cluster 3), and differentiation-committed clusters (clusters 0-1, 7), capturing combinatorial acetylation and methylation patterns of histone H3 (H3.1 and H3.2) associated with progression from progenitor-like states toward differentiation. Cluster 3 exhibits increased abundance of highly methylated H3 proteoforms relative to other clusters. Specifically, cluster 3 is characterized by acetylation at H3K4 with co-existing monomethylation at K9 or at lysine residues K27, K36, or K79 (*e.g*., H3K4acK9me1; H3K4acK27me1; H3K4acK36me1; H3K4acK79me1), consistent with a differentiating, transcriptionally active chromatin state (Supplemental Fig. S19, *A* and *B*) (88, 89, 90, 91).

Cluster 0 shows the abundance of H3 modified by acetylation at K4 and K9 together with dimethylation at K79 or at lysine residues K9, K27, or K36 (H3K4acK9acK79me2; H3K4acK9acK9me2; H3K4acK9acK27me2; H3K4acK9acK36me2), consistent with sustained transcriptional activity characteristic of differentiation-committed cell states. The presence of H3K79me2 and H3K36me2, which are established during transcriptional elongation, further supports an actively transcribed chromatin configuration (Supplemental Fig. S19*C*) (92, 93, 94, 95). Cluster 1 is enriched in H3 proteoforms characterized by trimethylation at K4 (H3K4me3) together with monomethylation at K9 or at lysine residues K27, K36, or K79 (H3K4me3K9me1; H3K4me3K27me1; H3K4me3K36me1; H3K4me3 K79me1), consistent with an active chromatin configuration associated with K4 trimethylation and a poised state reflected by K9 monomethylation (Supplemental Fig. S20*A*) (88, 96).

Cluster 7 exhibits increased abundance of H3.1 modified by acetylation at K4 (H3K4ac) together with dimethylation at K9 or at lysine residues K27 or K79 (H3.1K4ac K9me2; H3.1K4ac K27me2; and H3.1K4ac K79me2), which represents an accessible yet transcriptionally constrained cell state (Supplemental Fig. S20*B*) (86, 94). Consistent with this, downstream effect of higher-order methylation at K9 (H3K9me2/me3) is generally considered transcriptional silencing. However, mitosis-specific histone phosphorylation such as H3S10p was not observed in our current *snPiMS* dataset. This likely reflects the low fraction of mitotic nuclei in asynchronous keratinocyte populations analyzed in this study, as well as the challenges associated with detecting transient, low-stoichiometry phosphorylation in intact proteoform measurements. UMAP feature-expression plots further visualize the relative abundance of these H3 proteoforms corresponding to the differentiation states across the clusters are shown in Figure 5, *E*–*G*.

Across clusters, H3K27 and H3K36 methylation patterns reveal substantial proteoform heterogeneity associated with keratinocyte differentiation, with diverse combinatorial methylation states spanning mono-, di-, and higher-order modifications across multiple lysine residues. For example, clusters 0, 3, and 7 contain proteoforms such as H3K23acK36meK27me2, while cluster 8 exhibits higher-order states such as H3K4acK9acK36me2K79me2. These patterns span both UD- and DF-associated clusters, indicating dynamic combinatorial methylation rather than a single dominant configuration. This distribution is consistent with the established interplay between H3K27 and H3K36 methylation, where H3K36 methylation antagonizes PRC2-mediated H3K27 spreading (97), and H3K27 methylation is broadly associated with transcriptional repression during epidermal differentiation (98, 99). Notably, a discrete H3K27me2K36me2 configuration is not prominently resolved, consistent with both biological heterogeneity and the limitations of intact-mass measurements without MS/MS fragmentation for precise site localization.

Cluster 4, which is enriched in UD keratinocyte nuclei, is characterized by strong expression of HMG-17 (*HMGN2*) (Supplemental Fig. S21), a nonhistone chromosomal protein with high affinity for nucleosomes that is associated with transcriptionally active chromatin (100, 101). Consistent with prior studies showing dynamic expression of *HMGN2* during development and differentiation (102, 103, 104), its enrichment supports a progenitor-like chromatin state. Cluster 4 also exhibits higher abundance of acetylated H2A/H2B variants (H2A1ac, H2A2Aac, H2B1Nac) and H2A.Z (Supplemental Fig. S22*A*). Differential roles of H2A.Z at active and repressed genes were shown in the self-renewal and differentiation of mouse embryonic stem cells (105). In addition, cluster 4 shows N-terminally acetylated SmD2 (small nuclear ribonucleoprotein, SNRPD2) which is a well-known regulatory switch for the spliceosome (106).

Cluster 7 is further characterized by the presence of N-terminally acetylated H2AX (H2AXNαac) (Supplemental Fig. S22*B*). Clusters 8 and 11 show enrichment of HMGN3, another nucleosome-binding protein indicating potential heterogeneity in chromatin organization across nuclear subpopulations. The clusters (8, 9, 10, 11) are not discussed in detail here, and the corresponding UMAP feature expression plots are provided in the (Supplemental Fig. S23–S25).

### Comparison of Bulk Histone PTM Profiles and Single-Nucleus Proteoform Signatures

To independently characterize histone PTMs between UD and DF keratinocyte states, we performed a Tier 3 MRM LC–MS/MS analysis (107) of histones isolated from bulk nuclei (∼1 × 10^6^ per condition). The extracted histones were propionylated, digested, and analyzed to compare endogenous histone PTM peptide abundances between differentiation states, followed by differential expression analysis (Supplemental Figs. S26, S27, Supplemental Tables S6 and S7).

Among the quantified histone peptides, several PTMs exhibited statistically significant differences (adjusted *p* < 0.05, |log_2_FC| > 1), consistent with the changes in histone proteoform landscape observed along the differentiation trajectory in the single-nucleus analysis. Notably, DF nuclei exhibited higher relative abundance of several differentiation-associated histone marks, including repressive modifications on histone H4 and H3, such as H4K20me2 (log_2_FC = −6.24), H3K27me2 (log_2_FC = −3.70), H3R49me2 (log_2_FC = −4.42), and H3 K79me2 (log_2_FC = −3.77), together with increased H3K14Ac (log_2_FC = −2.97). Consistent with prior studies demonstrating that H3K14 acetylation can mark active, poised, or primed promoters, the enrichment of H3K14ac observed here in DF nuclei likely reflects selective maintenance or priming of lineage-specific transcriptional programs in differentiation (108). Thus, while differentiation is accompanied by a global increase in repressive histone methylation, the concurrent presence of H3K14 acetylation supports continued, regulated transcription of genes in the differentiation state suggests a primed regulatory landscape that may support selective and rapid transcriptional activation of gene programs potentially including immune and stress-response pathways (109, 110).

On the other hand, UD nuclei show higher relative abundance of several progenitor enriched/cell-cycle coupled histone marks, including PTMs on histone H4 and H3, such as H4K20me1 (log_2_FC = 7.94), H3K9me2 (log_2_FC = 5.3), and H3K36me2 (log_2_FC = 1.61). The higher abundance of H4K20me1 which is targeted to the newly synthesized histone H4 during S/G2-M phases and coupled to cell-cycle progression and differentiation (111). The presence of H3K9me2, a repressive epigenetic modification that represents one of the most abundant heterochromatin marks and has been reported to span large chromatin domains in DF cells (112, 113), suggests regulated transcriptional constraint rather than terminal silencing. Together, these proteoform signatures indicate that UD nuclei retain proteoform features coupled to the cell-cycle, reflecting a progenitor-like chromatin state prior to commitment to a program of differentiation.

## Discussion

This study establishes *snPiMS* as a new platform for probing dynamic changes in histone proteoform landscapes to probe human nuclei. Integration and normalization of >17,000 nuclei from UD and DF keratinocytes yielded 12 proteoform-defined nuclear clusters that likely represent distinct cell states. The distribution of nuclei across clusters ranging from UD enriched progenitor states (cluster 4), through intermediate and cell-cycle associated states (5), to DF enriched states (cluster 3), early differentiating clusters (0-2), and late differentiation states (cluster 7), delineates a continuous, proteoform-governed trajectory from cycling basal progenitors to terminally DF keratinocytes. This trajectory reflects a progressive chromatin compaction, histone variant exchange, and proteoform remodeling underlying epidermal lineage specification.

Similar to single-cell transcriptomic approaches, *snPiMS* resolves cellular heterogeneity and differentiation-associated states at single-nucleus resolution. However, unlike scRNA-seq, which captures dynamic gene expression and enables temporal inference, proteoform measurements represent endpoint molecular states and do not intrinsically encode directionality. Pseudotime and trajectory inference approaches (114, 115, 116, 117, 118) in single-cell transcriptomics rely on well-defined marker genes and marker expression gradients to order cells along a differentiation axis, as demonstrated in keratinocyte scRNA-seq studies using canonical markers such as *KRT5*, *KRT14*, *KRT1*, *KRT10*, *IVL*, and *FLG* (11, 119, 120, 121, 122). In contrast, proteoform-resolved measurements do not provide equivalent directional markers or monotonic trajectories. In our *snPiMS* analysis, histone proteoforms detected as combinatorial, and context-dependent states, with multiple co-occurring PTMs that do not follow a strictly ordered sequence. Accordingly, proteoform-defined clusters are best interpreted as reflecting phenotypic similarity and cellular state heterogeneity rather than explicit temporal ordering. Future integration with time-resolved measurements or multimodal alignment with transcriptomic data will offer new avenues for extending proteoform-based analyses toward temporal modeling.

Unlike bulk analyses that report population-averaged signals, *snPiMS* enables direct measurement of intact proteoforms at the single-nucleus level, revealing cell-to-cell variability in histone modification patterns that is otherwise obscured in bulk proteomics measurements. This enables identification of intermediate and transitional nuclear states along the differentiation continuum that are not captured by bulk top-down or bottom-up proteomic approaches. A central finding of this study is the ability of *snPiMS* to capture the progressive methylation on H4K20 with enrichment of H4K20me1 in cluster 5 and H4K20me2 in cluster 2, respectively, of cell-cycle progression and early differentiation in epidermal keratinocyte differentiation. These modifications are targeted to newly synthesized and unmethylated H4 which is deposited in chromatin during S phase and then monomethylated over mid-G_2_ to the mitosis followed by early G_1_ phase (82, 83). The resulting monomethylated H4 becomes dimethylated and trimethylated progressively from mid-G_2_ to G_1_ of the next cell cycle.

Similarly, histone H3 proteoforms delineate distinct clusters across keratinocyte differentiation, with cluster-specific proteoform markers reflecting active, poised, and repressed configurations. Differentiating states are enriched in combinatorial proteoforms characterized by H3K4 acetylation with monomethylation at K9, K27, K36, or K79, (*e*.*g*., H3K4acK9me1) consistent with transcriptionally active chromatin. Differentiation-committed states exhibit dual acetylation (H3K4acK9ac) together with dimethylation at K79, K27, K9, or K36, (*e*.*g*., H3K4acK9acK79me2) reflecting sustained transcriptional activity and elongation-associated chromatin (123). Additional states marked by H3K4me3 with co-occurring monomethylation (*e.g*., H3K4me3K9me1), consistent with an active chromatin configuration associated with K4 trimethylation and a poised state reflected by K9 (88, 96). Clusters enriched with H3K4 acetylation with higher-order methylation at K9 or K27 (*e*.*g*., H3K9me2/me3, H3K27me2) is indicative of accessible yet transcriptionally constrained or repressed chromatin states (130, 131).

Collectively, these patterns highlight a continuum of combinatorial histone H3 modification states underlying chromatin remodeling during epidermal differentiation. Across these states, H3K27 and H3K36 methylation do not converge on a single configuration such as H3K27me2K36me2, instead form diverse combinatorial landscapes, consistent with their known functional interplay between H3K27 and H3K36 methylation status. H3K36 methylation antagonizes PRC2-mediated H3K27 methylation spreading (particularly H3K27me2/me3), while H3K27 methylation is broadly associated with transcriptional repression during epidermal differentiation. (97). Together, these findings support a model in which H3K27 and H3K36 methylation exist in dynamic and heterogeneous combinatorial states, rather than as a single dominant configuration (98, 99).

Beyond histones, nonhistone chromatin-associated proteins further distinguish cell states, with enrichment of HMG-17 and acetylated H2A/H2B variants in UD nuclei and features such as H2AX acetylation in DF states, reflecting proteoform presentations along the lineage trajectory. Inclusion of ribosomal proteins in clustering analysis introduced strong nonhistone signals (*e*.*g*., RPL28, RPL30, RPL37A, RPS16) enriched in UD-associated clusters, which reduced clustering specificity for nuclear proteoform landscape. Although overall biological trends were preserved, these results support our strategy of restricting analyses to nuclear-enriched proteoforms to enhance the resolution of cell-states.

Importantly, these transitions are resolved here at the level of intact proteoforms in single nuclei, providing molecular evidence of cell cycle-coupled epidermal keratinocyte differentiation. Probing these transitions from UD stem cells toward postmitotic, molecularly distinct, and DF cells often spatially distant is very important in tissue regeneration such as in human skin epidermis (124). Recently, Cockburn *et al.* (125) showed that keratinocyte differentiation as a continuum of transcriptional changes whereby basal cells transitioned through a sequential upregulation of differentiation genes and downregulation of typical basal cell stemness genes. By blocking proliferative capacity of K10^+^ basal cells, differentiation-committed cells remained capable of dividing to produce daughter cells fated to further differentiate, demonstrating that differentiation is temporally uncoupled from cell cycle exit (11, 126, 127).

The ability of *snPiMS* to resolve the continuum of states in the epidermal keratinocyte differentiation and the associated histone proteoforms across thousands of nuclei represent a significant advance for single-cell proteomics. This trajectory reflects progressive and regulated epidermal differentiation defined by the histone proteoform landscape measured in single nuclei. However, this new platform yet requires more study and improved proteome coverage to accurately assign proteoform signatures to cell states in such complex mixtures. Beyond increasing the depth and number of proteoforms detected, isolating nuclei from tissue with difficult-to-isolate cell types is another avenue forward.

## Conclusion

This study demonstrates that *snPiMS* enables proteoform-resolved characterization of histone modification patterns in human keratinocyte differentiation, revealing combinatorial modifications at the single-molecule level and capturing heterogeneity across individual nuclei beyond what is typically resolved by genomics or immunostaining. Using *snPiMS*, we achieved high-throughput proteoform profiling of ∼50 nuclei per hour, enabling large-scale mapping of chromatin-state heterogeneity across more than 17,000 nuclei. *snPiMS* revealed proteoforms bearing both activating and repressive modifications within individual nuclei, defining chromatin plasticity as a hallmark of epidermal lineage progression. The resulting proteoform-defined clusters delineate a cell cycle-linked differentiation continuum, wherein proliferative S/G_2_-M phase progenitors transition from acetylation-dominant, transcriptionally active states to methylation-enriched, repressive configurations characteristic of terminal differentiation. Progressive methylation of histone H4K20me1 was found and validated as elevated in more proliferative UD states. Together, these datasets position keratinocyte differentiation as a continuous, proteoform-mediated chromatin trajectory rather than a discrete switch. Collectively, this work advances a new, highly scalable platform for single-nucleus proteomics and establishes a foundation for future investigations into cell typing, chromatin dysregulation, and single-cell biology.

## Data availability

All data generated in this study (.raw files of the s*nPiMS* experiments and processed single-nucleus DMT files) have been deposited and are available in the MassIVE repository (https://massive.ucsd.edu/) under the identifier MSV000100310 [https://doi:10.25345/C5G15TQ9S]. In addition, all raw files from both top-down and bottom-up proteomics experiments conducted in this study have been deposited to the same repository to ensure full transparency and data accessibility.

## Supplemental data

This article contains supplemental data(19 , 62 ,63 ,107, 128, 129, 130, 131, 132, 133, 134, 135, 136).

## Conflict of interest

The authors declare the following competing financial interest(s): N. L. K. and J. O. K. report a conflict of interest with I^2^MS technology, being commercialized by Thermo Fisher Scientific.
