# Supplementary material for: Proteoform Detection Across 17,000 Single Nuclei From Differentiating Skin Keratinocytes
Source: Mol Cell Proteomics. 2026 Jul 14;25(8):101621. doi: 10.1016/j.mcpro.2026.101621 (PMC13487837; doi:10.1016/j.mcpro.2026.101621)
Supplement: Supplementary file 1 [file mmc1.docx]

Proteoform Detection across 17000 Single Nuclei from Differentiating Skin Keratinocytes

Vijaya Lakshmi Kanchustambham^1^, Indira Pla^1^, Tian Xu^1^, Pei Su^2^, Nickolas P. Fisher^1^, Michael A.R. Hollas^2^, Paulina Markiv^3^, Uday Agarwal^1^, Monika Lysakowska^1^, Jared O. Kafader^1,2^, Xiaomin Bao^3^*, Neil L. Kelleher^1,2,4^*

**Supplemental Materials**

**Table of Contents**

Nuclei fixation and wash protocol……………………………………………………………………….....S-3

Bulk top-down proteomics analysis………………………………………………………………….……. S-3

Bulk bottom-up proteomics analysis of histone PTMs…………………………………………………......S-5

**Fig. S1.** Keratinocyte differentiation/nuclei isolation from neonatal foreskin epidermis ……………..…...S-8

**Fig. S2.** Targeted *snPiMS* workflow ………….…………………………………...………………….……S-9

**Fig. S3**. Real-time acquisition of *snPiMS* analysis of single nuclei ………………………………….…...S-10

**Fig. S4.** Single-Cell feature selection using MATLAB peak picking algorithm………….......…….....….S-11

**Fig. S5**. Single nuclei I^2^MS true mass-domain spectrum generation……………………….………….….S-12

**Fig. S6.** Experimental workflow of top-down proteomics database generation.………….…………...….S-13

**Fig. S7.** Panther classification of proteins.………......................................................................................S-14

**Fig. S8.** Intact Mass Tag (IMT) search analysis of proteoforms at MS1 level...………….……………....S-15

**Fig. S9.** IMT derived proteoform IDs of *snPiMS* data in the mass range 5-25 kDa...................................S-16

**Fig. S10.** Data analysis and processing workflow of s*nPiMS*………………………………….....S17

**Fig. S11.** Analytical workflow for data processing and Seurat clustering analysis. ……………………...S19

**Fig. S12.** Assessment of batch effects and data integration. ……………………………………………...S20

**Fig. S13.** Bright-field images of undifferentiated (UD) and differentiated (DF) nuclei before and after

*snPiMS* analysis.…………………………………………………………………………………………..S21

**Fig. S14.** Wash protocol optimization prior to *snPiMS* data acquisition………….………….............…...S22

**Fig. S15.** Effect of ribosomal proteoforms on clustering analysis…………………………………….…..S23

**Fig. S16.** Aggregated I^2^MS Spectra of clusters representing ribosomal proteoforms in the mass range

5-11 kDa………………………………………………………………………………………………….S24

**Fig. S17.** Aggregated I^2^MS Spectra of clusters representing ribosomal proteoforms in the mass range

11-16 kDa…………………………………………………………………………………………………S25

**Fig. S18.** Tandem-MS/MS fragmentation spectra of mono- and di- methylated histone 4 …..………....S-26

**Fig. S19**. Tandem-MS/MS fragmentation spectra of histone H3.2 ……………………………….……..S-27

**Fig. S20.** Tandem-MS/MS fragmentation spectra of histone H3.1………………………………..……..S-28

**Fig. S21.** Tandem-MS/MS fragmentation spectra of high-mobility group (HMG) family ……………...S-29

**Fig. S22.** Tandem-MS/MS fragmentation spectra of histone H2A variants…………..……………...….S-30

**Fig. S23.** UMAP plots for proteoform defined clusters (0-3, 5-8) in differentiation…………..………...S-31

**Fig. S24.** UMAP plots for proteoform defined clusters (4) in differentiation…………..…………….....S-32

**Fig. S25.** UMAP plots for proteoform defined clusters (9-11) in differentiation…………..……….…...S-33

**Fig. S26**. MRM based LC-MS/MS analysis of bulk nuclei of DF and UD cells….……………..…..…..S-34

**Fig. S27.** Differential expression of histone peptidoforms in bulk nuclei of DF and UD cells…………..S-35

**Table S1.** List of proteoforms used for I^2^MS data calibration………………………………….……...... S-36

List of Supplemental References……………………………………….…………….…….……….….....S-37

**Nuclei Fixation and Wash Protocol**

Briefly, 200 proof cold ethanol (−20°C) is diluted to 75/25 (v/v) ethanol/ water (-20 °C) and stored at –20°C for at least an hour prior to fixing the nuclei. We then gently add 4 mL of 75/25 (v/v) ethanol/ water (-20°C) drop wise directly into the chamber slide containing 1 mL of nuclei suspension in PBS to make up for a final volume of 5 ml of buffer. Swirl while adding every drop of ethanol mix to increase alcohol percentage uniformly and incubate the chamber slide for 5 min. at -20ºC. The buffer is vacuum aspirated out to dry and stored at -80ºC for further *snPiMS* analysis.

Prior to *snPiMS* experiments, we gently vacuum dry the slides taken out from –80°C and rinse sequentially with ethanol: water (90:10) for 30 s, and 100% ethanol for 30 s, respectively. The final washing is to remove any residual detergents and salts in the buffer, which causes the ion suppression.

**Top-down Proteomic Analysis (TDP) of Differentiated and Undifferentiated Nuclei**

**Sample preparation**

The UD and DF keratinocyte nuclei (1.1 million nuclei of each sample) were suspended in 50 µL of 20% v/v acetic acid and sonicated on ice with 40% amplitude for 10 seconds. Afterward, nuclei lysates were centrifuged at 14,000×g for 30 minutes at 4°C, and supernatants from centrifugation were collected for TDP.

**CZE-MS/MS and RPLC-MS/MS analyses for TDP of nuclei samples**

A combination of CZE-MS/MS and RPLC-MS/MS for TDP can potentially enhance proteome coverage. The complementarity of CZE and RPLC on proteoform identifications has been previously demonstrated by Drown *et al.* (1). Here, we performed TDP of differentiated and undifferentiated extracted nuclei evaluated in triplicate CZE-MS/MS and RPLC-MS/MS analyses to maximize nuclei proteoform identifications. For CZE-MS/MS analysis, a CESI 8000 Plus CE system (Sciex) was coupled to an Orbitrap Exploris 480 spectrometer (ThermoFisher Scientific) via an in-house-constructed electrokinetically pumped sheath-flow CE-MS nanospray interface (2, 3). A glass emitter (orifice size: 30-35 µM) on the interface was filled with sheath buffer (0.2% v/v formic acid and 10% v/v methanol) to produce electrospray at 2.2 kV. A one-meter LPA-coated capillary (50 µm I.D., 360 µm O.D.) was used for CZE separation. Fifty nL of sample was loaded into the capillary by applying a pressure of 5 psi for 11 seconds. The background electrolyte was 20% v/v acetic acid, and the separation voltage was 30 kV.

For RPLC-MS/MS, the separation was performed on the UltiMate 3000 LC system (Thermo Fisher Scientific). The LC was connected to the Orbitrap Exploris 480 spectrometer using a custom nano-electrospray interface. Before LC analysis, the samples remaining from CE experiments were diluted 20 times in a solution (mobile phase A) containing 0.2% v/v formic acid and 5% v/v ACN. 2 µL of each sample was loaded into an RPLC capillary column (75μm I.D., 25 cm length, PLRP-S beads, 5 μm particle size, 1000 Å pore size). LC mobile phase A was 0.2% v/v formic acid and 5% v/v ACN and mobile phase B was 0.2% v/v formic acid and 95% v/v ACN. RPLC gradient was maintained at 5% B from 0 to 10 minutes, and then was processed from 5% B to 15% B in 2 minutes, from 15% B to 35% B in 58 minutes, from 35% B to 95% B in 5 minutes, and then stayed at 95% B for 15 minutes at a flow rate of 500 nL/min.

The same mass spectrometry settings were applied for both CZE and RPLC approaches. The source temperature was 320°C and RF lens was 200%. The data was collected in DDA mode. For the full MS scan, the resolution was set to 120,000 (at m/z of 200), the m/z range was 500-2500, the normalized AGC target was 200%, the maximum injection time was set to “Auto”, and the loop count was 5 (top 5). Fragmentation was performed with an isolation window of 2 m/z. HCD collision energy was 32 %. MS/MS data was acquired at a resolution of 60,000 (at m/z 200), m/z range of 300-2000, normalized AGC target of 1000%, and “Auto” maximum injection time. The ion intensity threshold was set to 5e3 and the dynamic exclusion window was 60 s.

**Database Search**

The raw files collected above were processed using the publicly available TDPortal workflow (Code Set 4.0.0; <https://portal.nrtdp.northwestern.edu>), which performs intact mass inference, spectral deconvolution, and proteoform identification. The data were searched against the human proteome database derived from UniProt Swiss-Prot (June 2020 release) supplemented with curated histone sequences and common histone post-translational modifications. The proteoform identifications were filtered using 1% false discovery rate (FDR) estimated using a conservative, and context-dependent strategy to maintain 1% FDR at multiple molecular levels, including proteoform spectral match (PrSM), protein, isoform, and proteoform.

Other search parameters include: Empirical P-Score: False; Filter by FDR: True; Create SAS Input Sheet for Quant: True; Precursor Resolution: High Resolution; Fragmentation Type: Auto; Include ProSight Error Tolerant Search: False. The TDPortal workflow generated a high-confidence proteoform reference library that was used for downstream intact mass tag search (IMT) analysis and annotation of single-nucleus PiMS datasets.

**Bottom-Up Proteomic Analysis of Differentiated and Undifferentiated Nuclei**

**Sample Preparation**

**Histone Extraction and Propionylation**

For the PRM LC–MS/MS assay (Tier 3) (4), histones isolated from bulk UD and DF nuclei (~1 × 10⁶ per condition) were propionylated, digested, and analyzed to comparatively assess endogenous histone PTM peptide abundances, without the use of isotopically labeled internal standards. Histones were recovered from bulk nuclei samples (~1 × 10⁶ nuclei per condition) isolated from differentiated (DF) and undifferentiated (UD) keratinocytes following previously published protocols (5, 6). Extracted histones were subjected to propionylation prior to enzymatic digestion to derivatize lysine residues and improve chromatographic resolution of histone peptides. The resulting peptides were analyzed using a PRM LC–MS/MS workflow corresponding to Tier 3.

Briefly, Histones were extracted with five volumes of 0.2 M sulfuric acid for 60 minutes at room temperature. Following centrifugation at 4000×g for 5 minutes, trichloroacetic acid was added to the supernatant to a final concentration of 20% (v/v). Precipitated histones were pelleted at 10,000×g for 5 minutes, washed once with acetone containing 0.1% HCl, then twice with acetone. Histones were air dried, then resuspended in 10 μl of 100mM ammonium bicarbonate for derivatization by propionic anhydride, and digested with trypsin, as described previously (7-11). Samples were propionylated with 30 μl of freshly prepared 1:3 (v/v) propionic anhydride to isopropanol and adjusted to pH 8 with ammonium hydroxide. Samples were derivatized for 60 minutes at 50^o^C, dried by vacuum centrifugation, then digested overnight with 500 ng of trypsin at 37°C. Peptides were dried by vacuum centrifugation then underwent an additional round of propionylation as described above. Peptides were resuspended in 50μl of 0.1% aqueous trifluoroacetic acid then transferred to glass auto-sampler vials prior to mass spectrometry analysis.

**Nano-LC-MS Data Acquisition**

Multiple reaction monitoring (MRM) was performed on a TSQ Altis triple quadrupole (QqQ) mass spectrometer coupled to an UltiMate 3000 Dionex nano HPLC system (both Thermo Fisher Scientific). Peptides were loaded onto a C18 trap column (75 µm × 2 cm, Acclaim PepMap 100) then separated using an PicoChip analytical column (75 µm × 10 cm, ProntoSIL C18-AQ, 3 µm, 200 Å resin, New Objective PicoChip). Peptide separation was achieved using an increasing solvent gradient of mobile phase A (0.1% aqueous formic acid) and mobile phase B (95% acetonitrile and 0.1% aqueous formic acid) with conditions: 0 to 35% B at 300 nl/min flow rate over 45 minutes. The following QqQ settings were used across all analyses: electrospray voltage of 2.5 kV, argon collision gas pressure of 1.5mTorr, Q1 peak width of 0.7 FWHM, 2 second cycle time, and skimmer offset of 10 V. Modified and unmodified histone peptides monitored in the assay were selected based on previous reports (11, 12).

**Data analysis**

Targeted bottom-up LC–MS/MS data acquired in multiple reaction monitoring (MRM) mode were processed in Skyline using Savitzky–Golay smoothing (13). Raw mass spectrometry files were imported, and chromatograms were extracted for predefined peptide transitions corresponding to histone post-translational modifications (Supplementary Table S7). Chromatographic traces were smoothed using the Savitzky–Golay algorithm, followed by automatic peak detection and integration in Skyline. All integrated peaks were manually inspected to ensure correct peak assignment and to remove potential interferences or poorly defined signals.

Quantification was based on the integrated peak areas of selected fragment ion transitions for each targeted peptide. Relative abundances of histone modification states were calculated from peptide peak areas and further processed using in-house software to generate graphical representations and summary statistics. Final quantitative values represent the mean of three technical replicates, with variability reported as standard deviation.

**Fig. S1. Keratinocyte differentiation and nuclei isolation from neonatal foreskin epidermis.** **(A)** Isolation of primary cells from human neonatal skin epidermis tissue, the cell expansion in the progenitor state, and calcium chloride induced keratinocyte differentiation. **(B)** Nuclei extraction from progenitor keratinocytes (UD nuclei) using hypotonic buffer NEBA and NEBB. **(C)** Nuclei extraction from differentiated keratinocytes (DF nuclei) using hypotonic buffer NEBA and NEBB. *Panel A created with* [*BioRender.com*](https://www.biorender.com/)*.*

**Fig. S2.** **Targeted snPiMS workflow.** **(A)** snPiMS; **(B)** Chronogram profiles of single nuclei at fixed MS injection times; 250 ms (black), 500 ms (red), 750 ms (blue). **(C)** Total proteoform ion counts determined for acquisitions of targeted ~20 single nuclei (UD) for each MS injection times of 250 ms, 500 ms, 750 ms. **(D)** Total proteoform ion counts determined for acquisitions of targeted ~20single nuclei. (DF): for each MS injection times 250 ms, 500 ms, 750 ms.

**Fig. S3 High-throughput *snPiMS* analysis of single nuclei.** **(A)** Real-time acquisition of chronograms from the sampling of nuclei using 60%/40% acetonitrile/water and 5% acetic acid. **(B)** Expanded region of the *snPiMS* data in the chronogram from 600-700 min acquisition time. **(C)** Chronograms represent single and multiple nuclei ion extraction profiles (D) Liquid Probe rastering across multi-nuclei. **(E)** Liquid Probe rastering across single nuclei Scale bar; 50 µm.

**Fig. S4.** **Single-cell feature selection using MATLAB.** **(A)** I^2^MS data resulting from single chronograms of single nuclei, multiple nuclei, and chemical noise associated with the glass slide. **(B)** Processed individual ions were filtered into single-cell or multi-cell features. **(C)** Multi-cell features were carefully filtered out using an ion count threshold of 1,500 over the baseline 500, and a minimum distance of 350 µm between adjacent peaks the half-width-at-half-maximum of chronogram peaks for single cells not more than 250 µm.

**Fig. S5.** **Single nuclei I^2^MS mass-domain spectrum generation.** **(A)** Manual counting of single-cell features using QuPath 0.5.1. **(B)** Modeling single nuclei population based on spatial coordinates on the slide using MATLAB. **(C)** Selection of single nuclei features and individual ion scans for proteoform analysis. **(D)** Box-Whisker plots for the distribution of processed and charge assigned ions of single nuclei. **(E)** Single nuclei I^2^MS true mass-domain spectrum derived from aggregated single nuclei.

**Fig. S6.** **Experimental workflow of top-down proteomics database generation. (A)** protein extraction from bulk nuclei of differentiated (DF) and undifferentiated (UD) cell types using the extraction buffer, 20% acetic acid. **(B)** LC-MS/MS and CZE-MS/MS analysis of bulk keratinocyte nuclei using top-down proteomics. **(C)** Database search using TDPortal to generate a reference database of proteoform from DF and UD nuclei raw LC-MS/MS and CZE-MS/MS experimental data. Proteoform counts were 450 and 353 for UD and DF nuclei, respectively.

**Fig. S7.** **Panther classification of proteins.** **(A)** Proteins identified in DF nuclei. **(B)** Proteins identified in UD nuclei.

**Fig. S8.** **Intact Mass Tag (IMT) search analysis of proteoforms at MS1 level.** **(A)** IMT search analysis of *snPiMS* aggregated data of DF single nuclei, tolerance ±3 ppm. **(B)** IMT search analysis of *snPiMS* aggregated data of UD single nuclei, tolerance ±5 ppm.

**Fig. S9.** **Intact Mass Tag (IMT) search analysis of proteoforms at MS1 level;** proteoform identifications were annotated to the I^2^MS data in the mass range 5-25 kDa (shown here are aggregated data from the ~8000 DF single nuclei). Proteoforms identified are ribosomal subunit proteins, core histone proteins, and high mobility group nucleosomal binding domain (HMGN) family proteins.

**Fig. S10. Data analysis and processing workflow of the single nucleus proteoform imaging mass spectrometry *(snPiMS).***

**(1)** nano-DESI–I²MS scanning enables proteoform extraction from drop-casted single nuclei via a liquid bridge; extraction profiles (chronograms) from individual nuclei are indicated by purple arrows.

**(2)** Selection of single-nucleus features and acquisition of individual ion scans for proteoform analysis. A real-time total ion chronogram is shown, with red bars indicating positions of ~1,000 single-nucleus chronograms detected in the dataset.

**(3)** Aggregated I²MS spectrum generated from ~1,000 single nuclei in a single run. Spectra from individual nuclei were processed using STORIboard (Proteinaceous).

**(4–5)** Proteoform identification via intact mass tag (IMT) matching, where isotopic distribution shape and mass accuracy of the features from the aggregated snPiMS spectrum are compared to theoretical proteoforms derived from a custom top-down LC-MS/MS database derived from the bulk undifferentiated and differentiated nuclei.

**(6)** Single-nucleus proteoforms (snProteoforms) and proteoform assignment scores (PAScores). Representative data from a targeted nucleus#1035 (upper panel, black spectrum; 500 ms injection time; HCD gas pressure 0.5) are shown. Theoretical isotopic distributions (purple bars), experimental spectra (black trace), and assigned snProteoforms (red dots with droplines) are illustrated for HMGN2 (left) and H2A.Z (right). The number of matched ions, total ions per proteoform, and PAScores are indicated. Each dot represents an individual ion.

**(7)** Seurat-based clustering of nuclei using proteoform intensities assigned via IMT matching, filtered at 10% FDR against the top-down LC–MS/MS proteoform library. scAPP computes proteoform intensities and PAScores on a per-nucleus basis, analogous to quantitative feature matrices generated in single-cell omics workflows.

**Fig. S11.** **Analytical workflow for data processing and Seurat clustering analysis.** Schematic overview of the computational pipeline for single nucleus proteoform analysis. Raw snPiMS data are processed to extract proteoform intensities and assignment scores (PAScores) at the single-nucleus level, followed by data normalization and scaling. Dimensionality reduction is performed using principal component analysis (PCA), followed by visualization using UMAP or t-SNE. Nuclei are then clustered using the Seurat framework, and cluster-specific proteoform markers are identified for downstream biological interpretation and analysis.

**Figure S12. Assessment of batch effects and data integration.** PCA and UMAP embeddings of all nuclei colored by **batch** **(A, B, C)** and **cell type** **(D).** Batch labels are well mixed across the manifold, while biological labels (DF vs UD) remain coherent, indicating that the global structure is driven by biological variation rather than sample preparation or acquisition batch effects.

**Fig. S13. Bright-field images of undifferentiated (UD) and differentiated (DF) nuclei before and after *snPiMS* analysis.** **(A)** Representative images acquired prior to washing and *snPiMS* analysis, *Inset* showing nuclei (red arrows, rectangle), and the surrounding matrix (blue arrows). **(B)** Images acquired after washing and following *snPiMS* analysis, demonstrating removal of matrix background and retention of isolated nuclei. Images were collected using a 4× objective; scale bar, 1000 µm.

**Fig. S14. Wash protocol optimization prior to *snPiMS* analysis. (A)** Chamber slides washed with 100% ethanol for 30 s prior to *snPiMS* analysis **(B)** Chronograms of single nuclei profiles; **(C)** Corresponding MS spectra showing the individual ions with interfering background peaks at RT 0.87 min. **(D)** Chamber slides washed subsequently with 90%/10% ethanol/water for 30 s, and 100% ethanol for 30 s prior to *snPiMS* analysis. **(E)** Chronograms of single nuclei profiles. **(F)** Corresponding MS spectra showing the individual ions with suppressed background peaks at RT 670.88 min.

**Fig. S15. Effect of ribosomal proteoforms on clustering analysis. (A)** Seurat clustering of snPiMS data into five clusters when ribosomal proteoforms are included in the reference library. **(B)** Cluster composition by cell type, showing the relative proportions of differentiated (DF) and undifferentiated (UD) nuclei within each cluster. **(C)** UMAP visualization of nuclei colored by cell type across clusters. **(D)** Butterfly plot illustrating differential expression of histone H4 proteoforms, highlighting H4K20me1 and H4K20me2 (purple arrows) between clusters 4 and 3. **(E)** Butterfly plot illustrating differential expression of histone H3 proteoform methylation marks between clusters 1 and 0. Representative plots (D, E) highlight cluster-specific proteoform markers associated with cell-cycle progression and differentiation, respectively.

**Fig. S16. Aggregated I^2^MS Spectra of clusters.** **(A)** Representation of ribosomal proteoforms in the mass range 5-11 kDa, differentiation enriched clusters 0, 1 **(B)** Aggregated I2MS Spectra of clusters. **(A)** Representation of ribosomal proteoforms in the mass range 5-11 kDa, progenitor and cell-cycle enriched clusters 2,3, and 4.

**Fig. S17.** **Aggregated I^2^MS Spectra of clusters.** **(A)** Representation of ribosomal, high mobility group nucleosome-binding domain-containing protein, and histone proteoforms in the mass range 11-16 kDa, differentiation enriched clusters 0, 1 **(B)** Representation of ribosomal, High mobility group nucleosome-binding domain-containing protein, and histone proteoforms in the mass range 11-16 kDa, progenitor and cell-cycle enriched clusters 2,3, and 4.

**Fig. S18.** **Top-down Proteomics LC-MS/MS fragmentation spectra of internally acetylated and methylated proteoforms of histone H4.** **(A)** MS2 spectrum characteristic of the proteoform PFR219365; (H4-αAcS1+AcK12+1MeK20), sequence coverage 34.7%. **(B)** MS2 spectrum characteristic of the proteoform PFR6994; ((H4-αAcS1+AcK12 2MeK20) sequence coverage 42.6%.

**Fig. S19.** **Top-down Proteomics LC-MS/MS fragmentation spectra of 3 proteoforms of histone H3.2.** **(A)** MS2 spectrum characteristic of the proteoform PFR170246; H3 (H3-AcK4 +1MeK9) sequence coverage 20.9%. **(B)** MS2 spectrum characteristic of the proteoform PFR414856; H3 (H3-AcK4 +1MeK36) sequence coverage 20.9%. **(C)** MS2 spectrum characteristic of the proteoform PFR946634; H3 (H3-AcK4+AcK9+2MeK79 sequence coverage 20.9%.

**Fig. S20.** **Top-down Proteomics LC-MS/MS fragmentation spectra of histone H3.1. (A)** MS2 spectrum characteristic of the proteoform PFR169956; H3 (H3-3MeK4 +1MeK9), sequence coverage 17.2%. **(B)** MS2 spectrum characteristic of the proteoform PFR170033; H3 (H3-AcK4 +2MeK9), sequence coverage 20.1%.

**Fig. S21.** **Top-down Proteomics LC-MS/MS fragmentation spectra of high-mobility group (HMG) family.** **(A)** MS2 spectrum characteristic of the protein HMGN2, PFR1006; sequence coverage 51.1%. **(B)** MS2 spectrum characteristic of the protein HMGN1, PFR1022; sequence coverage 26.5%. **(C)** MS2 spectrum characteristic of the protein HMG-I/HMG-Y, PFR3596; sequence coverage 21.9%.

**Fig. S22.** **Top-down Proteomics LC-MS/MS fragmentation spectra of selected histone variants.** **(A)** MS2 spectrum characteristic of the proteoform PFR1144; canonical form of H2A.Z, sequence coverage 30.2%. **(B)** MS2 spectrum characteristic of the proteoform PFR1427; canonical form of H2A.X, sequence coverage 14.9%.

**Fig. S23.** **Cluster-specific proteoform expression patterns revealed by *snPiMS*.** **(A–H)** UMAP visualization of nuclei highlighting the top proteoform features most highly expressed in each cluster (0–3, 5-81), derived from integrated single-nucleus proteoform imaging mass spectrometry (*snPiMS*) data. Each panel shows relative expression intensity of representative proteoforms within the corresponding cluster, revealing distinct chromatin modification patterns and proteoform signatures that define UD, differentiating, and DF keratinocyte states along the epidermal lineage trajectory.

**Fig. S24.** **Cluster-specific proteoform expression patterns from integrated *snPiMS*. (A-C)** UMAP maps showing proteoform features enriched in cluster 4; **panel A** (HMGN2), **B** (H2A1+aαS1+AcK6), **C** (H2AZ1), revealing distinct chromatin modification patterns and proteoform signatures that define UD, differentiating, and DF keratinocyte states along the epidermal lineage trajectory.

**Fig. S25.** **Cluster-specific proteoform expression patterns from integrated *snPiMS*. (A-D)** UMAP maps showing proteoform features enriched in cluster 9-11; **panel A** (H3.1-AcK4+2meK9+1meK79), **B** (H2B1B), **C** (H3.1AcK4+mek27), and **D** (H3.1-AcK4+AcK9+1meK36+2meK79) revealing distinct chromatin modification patterns and proteoform signatures that define UD, differentiating, and DF keratinocyte states along the epidermal lineage trajectory.

**Fig. S26.** **MRM-based quantitative bottom-up LC–MS analysis of bulk keratinocyte nuclei isolated from undifferentiated (UD) and differentiated (DF) cells.** Box plots show the differences in intensity (log2) between UD and DF for individual histone marks **(A-C)** H4 peptidoforms; H4:K20 me1, H4:K20me2, H4:K20me3. **(D-H)** H3 peptidoforms; H3:K9 me2, H3:K79me2, H3:K27me2. **(I)** H1 peptidoform (H1.4: K25me2). Statistical significance was determined using BH-adjusted *p* values.

**Fig. S27.** **Differential expression of histone peptidoforms in bulk nuclei of DF vs UD cells.** Volcano plot revealed 10 histone peptidoforms with significantly higher abundance in undifferentiated nuclei (UD, red circles) and 15 with significantly higher abundance in the differentiated nuclei (blue circles).

**Supplemental Table S1. List of proteoforms used for I^2^MS data calibration.**

| Proteoform |  | Modification | | | Theor. Mass (Da) | | Mono m/z (Da) | ppm Error |
| --- | --- | --- | --- | --- | --- | --- | --- | --- |
| \|P05204\|HMGN2_HUMAN  Non-histone chromosomal protein HMG-17 | | |  | N. term. Acetyl | | 9256.02 | 9257.02 | -0.76 |
| \|P62805\|H4_HUMAN  Histone H4 | | |  | N. term. acetyl | | 11271.35 | 11272.36 | 0.07 |
| \|P62805\|H4_HUMAN  Histone H4 | | |  | Monomethyl | | 11285.37 | 11286.37 | -1.3 |
| \|P62805\|H4_HUMAN  Histone H4 | | |  | di-methyl | | 11299.38 | 11300.39 | -0.5 |
| \|P33778\|H2B1B_HUMAN  Histone H2B type 1-B | | |  | N. term. Acetyl | | 13852.55 | 13853.56 | -0.14 |
| \|Q16778\|H2B2E_HUMAN  Histone H2B type 2-E | | |  | N. term. Acetyl | | 13822.54 | 13823.55 | -1.14 |
| \|Q16777\|H2A2C_HUMAN  HistoneH2A type 2-C | | |  | N. term. Acetyl | | 13890.81 | 13891.82 | -0.42 |
| \|P84243\|H33_HUMAN  Histone H3.3 | | |  | N. term. Acetyl | | 15318.5 | 15319.51 | 0.64 |

**Supplemental References**

1.	Drown, B. S., Jooß, K., Melani, R. D., Lloyd-Jones, C., Camarillo, J. M., and Kelleher, N. L. (2022) Mapping the Proteoform Landscape of Five Human Tissues. *Journal of Proteome Research* 21, 1299-1310

2.	Sun, L., Zhu, G., Zhang, Z., Mou, S., and Dovichi, N. J. (2015) Third-Generation Electrokinetically Pumped Sheath-Flow Nanospray Interface with Improved Stability and Sensitivity for Automated Capillary Zone Electrophoresis–Mass Spectrometry Analysis of Complex Proteome Digests. *Journal of Proteome Research* 14, 2312-2321

3.	Li, Y., Compton, P. D., Tran, J. C., Ntai, I., and Kelleher, N. L. (2014) Optimizing capillary electrophoresis for top-down proteomics of 30-80 kDa proteins. *Proteomics* 14, 1158-1164

4.	Carr, S. A., Abbatiello, S. E., Ackermann, B. L., Borchers, C., Domon, B., Deutsch, E. W., Grant, R. P., Hoofnagle, A. N., Hüttenhain, R., Koomen, J. M., Liebler, D. C., Liu, T., MacLean, B., Mani, D. R., Mansfield, E., Neubert, H., Paulovich, A. G., Reiter, L., Vitek, O., Aebersold, R., Anderson, L., Bethem, R., Blonder, J., Boja, E., Botelho, J., Boyne, M., Bradshaw, R. A., Burlingame, A. L., Chan, D., Keshishian, H., Kuhn, E., Kinsinger, C., Lee, J. S. H., Lee, S.-W., Moritz, R., Oses-Prieto, J., Rifai, N., Ritchie, J., Rodriguez, H., Srinivas, P. R., Townsend, R. R., Van Eyk, J., Whiteley, G., Wiita, A., and Weintraub, S. (2014) Targeted Peptide Measurements in Biology and Medicine: Best Practices for Mass Spectrometry-based Assay Development Using a Fit-for-Purpose Approach *<sup>	</sup>. *Molecular & Cellular Proteomics* 13, 907-917

5.	Neely, A. E., and Bao, X. (2019) Nuclei Isolation Staining (NIS) Method for Imaging Chromatin-Associated Proteins in Difficult Cell Types. *Curr Protoc Cell Biol* 84, e94

6.	Bao, X., Siprashvili, Z., Zarnegar, B. J., Shenoy, R. M., Rios, E. J., Nady, N., Qu, K., Mah, A., Webster, D. E., Rubin, A. J., Wozniak, G. G., Tao, S., Wysocka, J., and Khavari, P. A. (2017) CSNK1a1 Regulates PRMT1 to Maintain the Progenitor State in Self-Renewing Somatic Tissue. *Dev Cell* 43, 227-239.e225

7.	Sidoli, S., Bhanu, N. V., Karch, K. R., Wang, X., and Garcia, B. A. (2016) Complete Workflow for Analysis of Histone Post-translational Modifications Using Bottom-up Mass Spectrometry: From Histone Extraction to Data Analysis. *JoVE*, e54112

8.	Searfoss, R. M., Karki, R., Lin, Z., Robison, F., and Garcia, B. A. (2023) An Optimized and High-Throughput Method for Histone Propionylation and Data-Independent Acquisition Analysis for the Identification and Quantification of Histone Post-translational Modifications. *Journal of the American Society for Mass Spectrometry* 34, 2508-2517

9.	Zahn, E., Xie, Y., Liu, X., Karki, R., Searfoss, R. M., de Luna Vitorino, F. N., Lempiäinen, J. K., Gongora, J., Lin, Z., Zhao, C., Yuan, Z.-F., and Garcia, B. A. (2025) Development of a High-Throughput Platform for Quantitation of Histone Modifications on a New QTOF Instrument. *Molecular & Cellular Proteomics* 24

10.	Garcia, B. A., Mollah, S., Ueberheide, B. M., Busby, S. A., Muratore, T. L., Shabanowitz, J., and Hunt, D. F. (2007) Chemical derivatization of histones for facilitated analysis by mass spectrometry. *Nature Protocols* 2, 933-938

11.	Zheng, Y., Sweet, S. M. M., Popovic, R., Martinez-Garcia, E., Tipton, J. D., Thomas, P. M., Licht, J. D., and Kelleher, N. L. (2012) Total kinetic analysis reveals how combinatorial methylation patterns are established on lysines 27 and 36 of histone H3. *Proceedings of the National Academy of Sciences* 109, 13549-13554

12.	Zheng, Y., Thomas, P. M., and Kelleher, N. L. (2013) Measurement of acetylation turnover at distinct lysines in human histones identifies long-lived acetylation sites. *Nature Communications* 4, 2203

13.	MacLean, B., Tomazela, D. M., Shulman, N., Chambers, M., Finney, G. L., Frewen, B., Kern, R., Tabb, D. L., Liebler, D. C., and MacCoss, M. J. (2010) Skyline: an open source document editor for creating and analyzing targeted proteomics experiments. *Bioinformatics* 26, 966-968
